# Stress-induced premature senescence is associated with a prolonged QT interval and recapitulates features of cardiac aging

**DOI:** 10.7150/thno.70884

**Published:** 2022-07-04

**Authors:** Edoardo Lazzarini, Alessandra Maria Lodrini, Martina Arici, Sara Bolis, Sara Vagni, Stefano Panella, Azucena Rendon-Angel, Melissa Saibene, Alessia Metallo, Tiziano Torre, Giuseppe Vassalli, Pietro Ameri, Claudia Altomare, Marcella Rocchetti, Lucio Barile

**Affiliations:** 1 Cardiovascular Theranostics, Istituto Cardiocentro Ticino, Laboratories for Translational Research, Ente Ospedaliero Cantonale, Bellinzona, Switzerland.; 2 Department of Biotechnology and Biosciences, Università degli Studi di Milano-Bicocca, Milano, Italy.; 3 Department of Cell and Chemical Biology, Leiden University Medical Center, Leiden, Netherlands.; 4 Cellular and Molecular Cardiology, Istituto Cardiocentro Ticino, Laboratories for Translational Research, Ente Ospedaliero Cantonale, Bellinzona, Switzerland.; 5 Department of Cardiac Surgery Istituto Cardiocentro Ticino, Ente Ospedaliero Cantonale, Lugano, Switzerland.; 6 Faculty of Biomedical Sciences, Università della Svizzera Italiana, Lugano, Switzerland.; 7 Department of Earth and Environmental Sciences, Università degli Studi di Milano-Bicocca, Milano, Italy.; 8 Cardiovascular Disease Unit, IRCCS Ospedale Policlinico, Genova, Italy.; 9 Department of Internal Medicine, University of Genova, Genova, Italy.; 10 Institute of Life Science, Scuola Superiore Sant'Anna, Pisa, Italy.

**Keywords:** Induced pluripotent stem cell-derived cardiomyocytes, senescence, aging, heart

## Abstract

**Rationale:** Aging in the heart is a gradual process, involving continuous changes in cardiovascular cells, including cardiomyocytes (CMs), namely cellular senescence. These changes finally lead to adverse organ remodeling and resulting in heart failure. This study exploits CMs from human induced pluripotent stem cells (iCMs) as a tool to model and characterize mechanisms involved in aging.

**Methods and Results:** Human somatic cells were reprogrammed into human induced pluripotent stem cells and subsequently differentiated in iCMs. A senescent-like phenotype (SenCMs) was induced by short exposure (3 hours) to doxorubicin (Dox) at the sub-lethal concentration of 0.2 µM. Dox treatment induced expression of cyclin-dependent kinase inhibitors p21 and p16, and increased positivity to senescence-associated beta-galactosidase when compared to untreated iCMs. SenCMs showed increased oxidative stress, alteration in mitochondrial morphology and depolarized mitochondrial membrane potential, which resulted in decreased ATP production. Functionally, when compared to iCMs, SenCMs showed, prolonged multicellular QTc and single cell APD, with increased APD variability and delayed afterdepolarizations (DADs) incidence, two well-known arrhythmogenic indexes. These effects were largely ascribable to augmented late sodium current (I_NaL_) and reduced delayed rectifier potassium current (Ikr). Moreover sarcoplasmic reticulum (SR) Ca^2+^ content was reduced because of downregulated SERCA2 and increased RyR2-mediated Ca^2+^ leak. Electrical and intracellular Ca^2+^ alterations were mostly justified by increased CaMKII activity in SenCMs. Finally, SenCMs phenotype was furtherly confirmed by analyzing physiological aging in CMs isolated from old mice in comparison to young ones.

**Conclusions:** Overall, we showed that SenCMs recapitulate the phenotype of aged primary CMs in terms of senescence markers, electrical and Ca^2+^ handling properties and metabolic features. Thus, Dox-induced SenCMs can be considered a novel *in vitro* platform to study aging mechanisms and to envision cardiac specific anti-aging approach in humans.

## Introduction

It is estimated that by the year 2035, nearly one in four individuals will be 65 years of age or older, and this change in the world demographics will result in a large increase in the prevalence of age-related cardiovascular disabilities 1. Primary drivers of tissue damage in aging, such as oxidative stress, DNA damage, mitochondrial dysfunction, and metabolic dysregulation, play a role in inducing cellular senescence. The latter represents a cellular response to such stimuli, thereby being defined as an "antagonistic" hallmark of aging 2. The discovery that senescent cells aberrantly accumulate in aging tissues has substantiated the hypothesis that senescence itself can drive aging 2-4. Indeed, accumulating senescent cells secrete pro-inflammatory molecules triggering the senescence-associated secretory phenotype (SASP), with deleterious effects on the tissue microenvironment leading to age-dependent functional impairment 5, 6. Advanced age has been identified as one of the traditional risk factors for the development of aging-related cardiovascular disease (CVD) 7, increasing incidence of death, disability, and morbidity 8 with a high impact on the utilization of healthcare resources 9. Cardiomyocytes (CMs) enduring exogenous cellular insult may develop a stress-induced premature senescence (SIPS) phenotype, which recapitulates many cellular and molecular features as those undergoing natural aging process 10, 11. SIPS has emerged as possible link between long-term consequences of some pathological conditions such as sepsis 12 or anthracycline cardiotoxicity 13 and cardiovascular complications 14, 15. Nowadays, cellular mechanisms behind such unexplained cardiac abnormalities regardless of age, are not completely understood.

It has been shown that aging alters the pattern of electrical activation in the heart of overtly healthy older subjects, resulting in prolonged myocardial repolarization 16, thus increasing the risk of malignant ventricular arrhythmias and sudden death 17. Evidence in small animal model suggested that cardiac performance in aging heart is affected by electrical alterations at cellular level 18. Increase in the late Na^+^ current (I_NaL_) in senescent mouse CMs prolongs the action potential (AP) and influences temporal kinetics of Ca^2+^ cycling 18. However, whether this applies also for human cells is unknown. Therefore, studying the mechanisms underlying intracellular ionic balance in senescent human CMs, that constitute the vast majority of cardiac cell mass, 19 might pave the way for understanding the intricacies of age-related changes in the physiology of human heart 18, 20 and the increased susceptibility of the aged heart to injury 21.

Currently, the most used approaches to study cellular senescence are based on aging prone transgenic organisms 22. However, the cardiovascular phenotype of transgenic animals only partially mimics the changes occurring in humans. CMs derived from human induced pluripotent stem cells (iCMs) offer an unprecedented platform that overcomes not only species-specific limitations but also the technical difficulties of accessing human primary CMs as well as their limited lifespan in culture. This study aimed to develop an *in vitro* tool for understanding human cardiac senescence's molecular basis that may affect age-related CVDs. Based on extensive published data showing that doxorubicin (Dox) can induce senescence in neonatal murine CMs 23, cardiac progenitor cells (CPCs) 24, 25 and vascular smooth muscle cells 26, we used sub-apoptotic doses of Dox as an inducer of SIPS in human iCMs. We explored phenotypic, functional and metabolic properties of human senescent CMs (SenCMs) in comparison to untreated controls (iCMs). Moreover, our model elucidated some basic aspects of SIPS, which is well known to impact diastolic and systolic function following stressors such as myocardial infarction and cancer therapy-induced cardiotoxicity 27, 28. We compared our findings in human cells with electrophysiological changes occurring in CMs of naturally aged mice highlighting the importance of modelling senescence in human cells. By taking advantage of in-house biobank of human atrial appendage tissue specimens we correlated the expression of specific ion channels with age in heart tissue of small observational cohort of patients. Finally, some key markers of SIPS in CMs were validated by analyzing open-access heart-tissue transcriptomic data.

## Methods and Materials

### Data Source and Collection

Right cardiac atrial appendage tissue specimens were collected from patients who underwent surgical repair of heart valves and had no concomitant coronary artery disease. Patients gave written informed consent. Protocols used in this study were approved by local Ethical Committee for Clinical Research (Comitato Etico Cantonale, Bellinzona, Switzerland; Rif. CE 2923), and study was performed in accordance with the Declaration of Helsinki. The observational study for the expression of KCNH2 gene was performed on atrial appendage tissue from 13 consecutive enrolled male patients between Feb 2018 and Nov 2019. Mean age was 68±8.7 (55-78). All experiments involving animals conformed to the guidelines for Animal Care endorsed by the University of Milano-Bicocca (project 29C09.N.5TB) and to the Directive 2010/63/EU of the European Parliament on the protection of animals used for scientific purpose.

Data for single nuclei transcriptomic analysis were obtained from Human Heart Atlas database (https://www.heartcellatlas.org/), a specific section of the Human Cell Atlas initiative (https://data.humancellatlas.org/) 29.

### Generation and characterization of human Induced pluripotent stem (iPS) cells

iPS cells were obtained by the reprogramming of adult stromal cardiac-specific mesenchymal cells (cMSC). Somatic cells were derived as cellular outgrowth from the explants using an *ex vivo* primary tissue culture technique, as previously described 30-32. Briefly, atrial tissue was rinsed with phosphate buffer saline (PBS) and cut into small pieces that were placed into a 100-mm cell culture dish (Corning). To facilitate cell outgrowth, atrial tissue was treated with Trypsin/EDTA (SIGMA Life Science) for 2-3 min. Tissue pieces were plated on 0,02% gelatin and cultured in IMDM medium (Iscove's Modified Dulbecco's Medium) supplemented with 20% fetal bovine serum (FBS) and 1% penicillin/streptomycin (all from Life Technologies). The culture medium was changed twice a week. After 25-30 days of culture, cMSC were enzymatically detached using Trypsin/EDTA and transferred to a gelatin-coated 35-mm dish (Corning, 2.5×10^5^ cells/dish). After additional 48 hours, cMSC were transduced with the integration-free Sendai virus cocktail hKOS:hc-Myc:hKlf4 at a MOI of 5:5:3 (CytoTune-iPS 2.0 Sendai Reprogramming Kit, Thermo Fisher Scientific), as per manufacturer's instructions. The virus was removed after 24 h and the medium was changed daily in the next 7 days. A week after transduction, medium was changed to StemFlex (Thermo Fisher Scientific). Individual colonies with embryonal stem cells (ESC)-like morphology typically appeared after 25-35 days and were transferred manually into 12-well plates coated with Matrigel (hESC Qualified Matrix, Corning) and expanded. Established human iPS cell lines were maintained in 33-mm Matrigel-coated plates (Falcon), passaged with TrypLe solution (TrypLE Express Enzyme 1x, Thermo Fisher Scientific) and cultured in StemFlex medium.

### Directed differentiation of iPS cells into iCMs

Directed differentiation of human iPS cells into iCMs was performed via WNT signaling pathway modulation. Differentiation was initiated at 90% confluence in 12-well Matrigel-coated plates with a differentiation medium composed of RPMI 1640 supplemented with B-27 minus insulin (Thermo Fisher Scientific) with 4 μM CHIR99021 (Merck Millipore) for 48 h and subsequently 5 μM IWP4 (Merck Millipore) for 48 hours 33. The medium was changed to a maintenance medium composed of RPMI 1640 with B-27 plus insulin (Thermo Fisher Scientific) at day 7. Metabolic selection of CMs was performed using a selection medium composed of RPMI 1640 without glucose (Thermo Fisher Scientific), 0.5 mg/ml human recombinant albumin, 0.2 mg/ml L-ascorbic acid 2-phosphate, and 4 mM lactate (Sigma-Aldrich) from days 10 to 17. Afterwards, iCMs were cultured in maintenance medium at least to day 30 for further maturation.

### Mouse CMs isolation

Ventricular CMs were isolated from young (7 weeks) and old (18 months) mice (mCMs) as previously described with minor modifications 34. Rod-shaped, Ca^2+^-tolerant mCMs were used within 12 hours from dissociation.

### Doxorubicin-induced CM senescence

Optimal conditions to obtain a Dox-induced senescent phenotype were empirically determined by dose response experiments, exposing iCMs to Dox at concentration ranging from 0.2 µM to 5 µM. Briefly, spontaneously beating iCMs were enzymatically dissociated (Multi Tissue Dissociation Kit 3, Miltenyi Biotec) and then plated in Synthemax II-SC Substrate (Corning)-coated wells (5×10^4^ cells/cm^2^). After 24-48 hours, part of the wells had medium replaced with Dox-containing maintenance medium to obtain senescent-like cardiomyocytes (SenCMs). After 3 hours from Dox administration, all cells were washed twice with PBS and placed back in fresh maintenance medium.

### Senescence associated (SA)-β-gal staining

iCMs were stained for senescence-associated β-galactosidase (SA-β-gal) activity as previously described 35. Briefly, cells were washed twice with PBS, fixed with 2% formaldehyde and 0.2% glutaraldehyde in PBS, and washed twice in PBS. Cells were stained in X-gal staining solution (1 mg/ml X-gal, 40 mM/l citric acid/sodium phosphate, 5 mM/l potassium ferricyanide, 5 mmol/l potassium ferrocyanide, 150 mM/l NaCl, 2 mM/l MgCl_2_, pH 6.0). After 6 hours, cells were washed twice with PBS. For a sensitive determination of the total cell number, cells were counterstained with 1 µg/ml Hoechst 33342 (Molecular Probes). Stained cells were examined using Lionheart FX Automated Microscope (BioTek Instruments Inc., Winooski, VT, USA) and analyzed with Gen 5.0 software (Biotek Instruments).

### Cell viability

Cellular viability was assessed by double labeling of cells with 1 μM calcein-AM and 1.2 μM DRAQ7; total nuclei were stained with 1µg/ml Hoechst 33342 (Merck). Viable total and dead cells were counted using the Lionheart FX Automated Microscope (BioTek Instruments Inc., Winooski, VT, USA). Apoptosis was further evaluated by Terminal deoxynucleotidyl transferase dUTP nick end labeling (TUNEL) assay. CMs were treated and after 24hours fixed in 4%methanol-free Paraformaldehyde (PFA) for 15 min then processed with DEadEnd Fluorimetric TUNEL system (Promega) following manufacturer protocol. Total cells were stained with DAPI 1µg/ml (Merck).

Stained cells were acquired using Lionheart FX Automated Microscope (BioTek Instruments) and analyzed with Gen 5.0 software (BioTek Instruments).

### Immunocytochemistry

After treatment, iCMs were washed twice with PBS and then fixed for 5 min at RT using a PFA-4%/sucrose-2% solution. Fixed cells were incubated for 10 min with glycine 0.1 M and then washed with PBS for 5 min. Cells were then permeabilized with 0.3% Triton X (Triton X detergent, Sigma-Aldrich) in PBS for 30 min, followed by a 5-min wash with PBS. Cells were then blocked with 2% bovine serum albumin (BSA) (Merck) for 1 h at RT. Subsequently, they were incubated with PBS containing 0.1% Tween, 1% BSA, and the primary antibody overnight at 4°C. To assess cardiac differentiation, cells were stained using antibodies against Sarcomeric Actin (α-S-Actin) (Abcam 9465), cardiac Troponin I (cTnI, AbCam 47003), cardiac Troponin T (cTnT) (13-11 Thermo Fisher Scientific), Myosin light chain 2 atrial (MLC2a, Synaptic System 311011), and Myosin light chain 2 ventricular (MLC2v, Proteintech 10906) to assess presence of DNA breaks, cells were stained using antibodies against γ-H2AX (9718 Cell Signaling), to assess cytotoxicity cells were stained with cleaved Caspase-3 Ab (Cell Signaling Technologies 9664). To assess senescence induction cells were stained with P16 and P21 (Proteintech 10355) Antibodies.

### Nuclei were counterstained with DAPI 1µg/ml or Hoechst-33342 1µg/ml

EdU (1 µM) was supplied to the cells 24hours after Dox treatment. After further 48hours cells were fixed and EdU incorporation was revealed through Click-it kit (Thermo Fisher Scientific) following manufacturer protocol to measure active DNA synthesis. Stained cells were examined using Lionheart FX Automated Microscope (BioTek Instruments) and analyzed with Gen 5.0 software (BioTek Instruments).

### Real time PCR analysis

iCMs were washed twice with PBS and RNA was extracted by adding 1 mL of TRI-Reagent (Sigma-Aldrich) as per manufacture instructions. For reverse transcription, 500ng of RNA was reverse-transcribed using GoScript Reverse Transcription System kit (Promega) as per manufacture instructions. To perform real-time PCR, the following mix was used: 2 μL DEPC water, 5 μL SsoAdvanced Universal SYBR Green Supermix 2x (BioRad), 2 μL of cDNA diluted 1:50 in DEPC water, 0.5 μL primers forward 10mM, and 0.5 μL primers reverse 10mM. Amplification and detection of specific products were performed in triplicate using a CFX Connect™ Real-Time PCR Detection System (Bio-Rad Labs). The threshold cycle (Ct) of each gene was automatically defined and normalized to the geometric mean control housekeeping genes GAPDH and RPL27(ΔCt value). To compare gene expression levels among different treatments, ΔΔCt values in treated CMs were calculated as the differences between the ΔCt value in these groups and the ΔCt value in untreated CMs following Livak method 36. Primers sequences are shown in Supplementary Table 1.

### Western blot analysis

Total proteins were extracted by lysing cells and then heated at 95 °C for 5 min (except for SERCA2 and PLN detection) with Laemmli SDS sample buffer 6× containing: 0.375 M Tris-HCl pH 6.8, 12% SDS, 60% glycerol, 0.6 M DTT, 20% (v/v) beta-mercaptoethanol, 70.2% (w/v) bromophenol blue (VWR International LCC). Proteins were separated on 4-20% Mini-PROTEAN® TGX™ Precast Gel (Bio-Rad) (or 4-12% Bis-Tris Criterion BIO-RAD gels for SERCA2 and PLN detection) and transferred onto a PVDF membrane with a semi-dry transfer system (Bio-Rad). The membranes were blocked for 1 h with Intercept (TBS) Blocking Buffer (Licor) or milk and incubated with the primary Abs at 4 °C overnight (anti-p16, 1:1'000, Proteintech 10883; anti-p21, 1:1'000 eBiosciences 14-6715-81; anti-KCNH2, 1:200, Alomone APC-109; anti-P AMPK, 1:1'000, Cell Signaling 2537; anti-CAMKII, 1:2'000, Abcam 92332; anti-P CAMKII, 1:2'000, Abcam 171095; anti-SERCA2, 1:1000, N-19 Santa Cruz Biotechnology; anti-PLN 1:1000, 2D12, Abcam; anti-actin 1:5000, Merck). Membranes were then rinsed and incubated with appropriated fluorophore-conjugated secondary antibodies (Li-COR) at RT for 2 h. Subsequently, the membranes were rinsed then acquired and analyzed using the Odyssey CLx Detection System (LI-COR Biosciences).

### ATP/AMP ratio

iCMs were lysed by incubation with an ultrapure water buffer containing perchloric acid 2.5% for 10 min, followed by a freeze (-20 °C)-thaw cycle. The lysate was then incubated with KH_2_CO_3_ for 5 min, resulting in precipitate and CO_2_ formation. At the end of the reaction, the supernatant was centrifuged (18000 G for 15 min) to remove any precipitate. For ATP detection, the supernatant was incubated with an ultrapure water buffer containing (mM) 100 Tris, 50 glucose, 0.2 NADP^+^, 5 MgCl_2_, 0.27 Hexokinase/Glucose-6-Phosphate dehydrogenase. The increase in absorbance over time indicated the reduction of NADPH and it correlated with the ATP levels in the supernatant. For AMP detection, the supernatant was incubated with an ultrapure water buffer containing (mM) 100 Tris, 0.15 NADH, 0.2 ATP, 1 Phospo-Enol-Pyruvate, 5 MgCl_2_, 0.27 Pyruvate Kinase/Lactate dehydrogenase, 0.27 Adenilate Kinase. The decrease in absorbance over time indicated the oxidation of NADH and it correlated with the AMP levels in the supernatant.

### Reactive oxygen species (ROS) detection assay

iCMs were seeded in 96-well/clear bottom plates for fluorescent measurements. Cells were washed twice with PBS and then incubated at 37°C in the dark with the ROS staining buffer containing dihydroethidium (5 µM) (Thermo Fisher Scientific). After 30 min of incubation, designated positive control wells were treated with TBHT (100 µM). After 30 additional minutes, iCMs were gently washed with PBS. The fluorescence was measured using the Infinite M-series fluorescent plate reader (TECAN) using an excitation wavelength of 480-520 nm and an emission wavelength of 580-600 nm. To exclude interference of Dox intrinsic fluorescence, fluorescent values were subtracted of baseline-cells treated with Dox but not incubated with dihydroethidium.

### Mitochondrial Stress Test Assay

iCMs and SenCMs were harvested and seeded at 7.5×10^4^ cells/well in a Seahorse XF miniplate (Agilent). After 48h the maintenance medium were chanced with Seahorse XF RMI supplemented 1 mM pyruvate, 2 mM glutamine, and 10 mM glucose with and cells were then analyzed for Oxygen Consumption Rate (OCR) with Seahorse XFp instruments, following Cell Mito Stress Test (Agilent) manufacturer protocol. OCR was measured every 7 minutes, inhibitors concentration was Oligomycin 1µM, FCCP 0.5µM, Rotenone/Antimycin A 1µM. Basal respiration was measured as OCR level previous to Oligomycin treatment minus minimum OCR level after Rotenone/Antimycin A injection. ATP linked production was measured as Maximum OCR level after Oligomycin injection minus Basal Respiration. Normalization for cell number was obtained with Sulforhodamine B assay. Briefly at the end of the experiment cells were fixed in 10% Trichloroacetic acid for 1h. Cells were washed and incubated with 0.057% SRB solution for 30 minutes. Cells were washed in acetic acid and air dried. Remaining cell-bind SRB were eluted in Tris pH10.5 and absorbance measured at 510nm wavelength in Tecan Infinite 2000 reader. Analysis was performed with Wave software (Agilent).

### Transmission electron microscopy (TEM) acquisition and mitochondrial analysis

iCMs were cultured on glass coverslips, washed with PBS and incubated overnight in a fixative solution (2.5% glutaraldehyde in PBS) at 4°C. Cells were then washed with PBS and post-fixed in aqueous osmium tetroxide solution (1%). Next, cells were dehydrated in a series of ethanol solutions (50%-70%-90%-95%-100%), incubated in grated absolute ethanol/propylene oxide solutions and infiltrated in epoxy resin. Following removal of glass coverslip, ultrathin sections (50-70 nm) were stained with uranyl acetate and lead citrate solutions. Samples were observed with JEOL JEM-2100 Plus (JEOL, Italy) transmission electron microscope at 200 kV.

The images were analyzed using ImageJ to quantify the following morphological mitochondrial shape descriptors: mitochondria surface area, Feret's diameter (longest distance between any two points within a given mitochondrion), aspect ratio (major axis)/(minor axis) as the measure of the “length to width ratio”.

### JC10 assay

Designed positive control cells were incubated with maintenance medium containing CCCP (carbonyl cyanide m-chlorophenyl hydrazine) at 37 °C for 1 hour. All wells were then washed twice with PBS and incubated with the JC10 dye (Abcam) as per manufacturer instructions. After 45 min of incubation, the fluorescence was measured using the Infinite M-series fluorescent plate reader (TECAN) using an excitation wavelength of 490 nm and emission wavelengths of 520-570 nm.

### Microelectrode arrays (MEA)

Field potentials (extracellular recordings) of spontaneously beating clusters of iCMs were recorded at 37 °C using a 60MEA100/10iR-Ti-gr 64-electrode Microelectrode Arrays (MEA, Multi Channel Systems). iCMs were seeded in the Syntemax-coated MEA chambers (volume 500 µl) for at least 4 days before appropriate Dox treatment and recordings. The latter were performed in maintenance medium. For each time-point, measurements were taken 10 min after signals had reached a steady state value. The duration of field potentials (FPD), reflecting the electrical systole, was measured from the onset of the sharp positive deflection to the peak of the secondary slow deflection. This measurement is representative of the electrocardiographic QT interval. The rate corrected QT intervals (QTc) were calculated by applying Bazett's correction (QTc = QT/√RR). MEA data analysis was performed with MC Rack, MC Data Tool (both by Multi Channel Systems) and Clampfit 10.7 (Molecular Devices).

### Patch clamp measurements

iCMs and mCMs were clamped in the whole-cell configuration. During measurements, myocytes were superfused at 2 ml/min with Tyrode's solution containing 154 mM NaCl, 4 mM KCl, 2 mM CaCl_2_, 1 mM MgCl_2_, 5 mM HEPES/NaOH, and 5.5 mM D-glucose, adjusted to pH 7.35. A thermostated manifold, allowing for fast (electronically timed) solution switch, was used for cell superfusion. All measurements were performed at 35 ± 0.5°C. The pipette solution contained (unless otherwise specified) 23 KCl, 110 KAsp, 0.4 CaCl_2_, 3 MgCl_2_, 5 HEPES-KOH, 1 EGTA-KOH, 0.4 NaGTP, 5 Na_2_ATP, 5 Na_2_PC, pH 7.3. Membrane capacitance (C_m_) and series resistance were measured in every cell but left uncompensated. Signals were acquired with MultiClamp 700B amplifier (Molecular Devices) connected to Digidata 1550A (Molecular Devices) and analyzed via pClamp 10.6 (Molecular Devices).

### Electrical activity

Action Potentials (APs) were acquired in iCMs and mCMs in I-clamp mode at specific stimulation rates. In particular, to overcome the low expression of the inward-rectifier potassium current (I_K1_) in iCMs, *in silico* I_K1_ was injected through the Dynamic Clamp (DC) technique as previously reported 37. Briefly, APs, recorded from iCMs, were acquired at a sampling rate of 10 kHz into the computer memory to drive the numerical I_K1_ according to the Nygren et al 38. C_m_ and the estimated liquid junction potential (8 mV) were computed in generating numerical I_K1_; I_K1_ maximal conductance was set to 0.7 nS/µF, as required to bring iCM diastolic potential (E_diast_) near -80 mV, and kept constant for all experiments. The AP duration at 90% of the repolarization phase (APD_90_) and the E_diast_ were quantified. Delayed afterdepolarizations (DADs) were defined as diastolic depolarizations with amplitude >1mV and their incidence was evaluated. Beat-to-beat variability of repolarization was expressed as the short-term variability (STV) of APD_90_ (i.e., the mean orthogonal deviation from the identity line 39, 40, calculated as follows: STV = ∑〖(|〖APD〗_(n+1)-〖APD〗_n |/[n_(beats ) x √2] 〗) for 30 consecutive APs (nbeats) at steady-state level.

### Membrane currents

The rapid delayed rectifier K^+^ current (I_Kr_) was recorded as E-4031 (3 μM)-sensitive current by applying depolarizing steps from -40 mV to +40 mV in the presence of I_Ks_ and I_CaL_ blockers HMR1556 (1 µM) and nifedipine (1 µM) respectively. I_Kr_ I/V relationships were obtained by measuring tail currents at -40 mV. The late component of the Na^+^ current (I_NaL_) was isolated as TTX (2 μM)-sensitive current (I_TTX_) by applying slow voltage ramps (28 mV/s) from -100 mV to +40 mV. I_TTX_ at 0 mV was taken as representative of I_NaL_, while peak I_TTX_ value, occurring at more negative potentials, was assumed to reflect the Na^+^ 'window component' (I_Naw_) as previously described 41. Current densities (pA/pF) were obtained by normalizing current amplitudes to C_m_.

### Intracellular Ca^2+^ dynamics

Cytosolic Ca^2+^ and membrane current were simultaneously recorded in V-clamped iCMs loaded with Fluo4-AM (10 µM). iCMs were superfused at 2 ml/min with the Tyrode's solution added with 2 mM 4-aminopyridine and 1 mM BaCl_2_ to block K^+^ currents. In the pipette solution EGTA was decreased to 0.1 mM and 0.01 mM Fluo4-K^+^ salt was added. Fluorescence at the holding potential (-80 mV) was used as reference (F_0_) for signal normalization (F/F_0_) after subtraction of background. Ca^2+^ transients (CaT) and I_CaL_ were recorded during 300 ms steps to 0 mV following 50 ms step to -35 mV to inactivate Na^+^ channels. I_CaL_ was isolated as nifedipine (10 μM)-sensitive current; nmols of Ca^2+^ entering the cell (CaL influx) were quantified integrating I_CaL_ during the step. Sarcoplasmic reticulum (SR) Ca^2+^ content (CaSR) was estimated by both integrating the Na^+^/Ca^2+^ exchanger (NCX) current (I_NCX_) and measuring CaT amplitude elicited by caffeine (10 mM) pulse, obtaining comparable results.

### Ca^2+^ sparks

Spontaneous unitary Ca^2+^ release events (Ca^2+^ sparks) were recorded at room temperature in Fluo4-AM (10µM) loaded myocytes at resting condition. Tyrode bath solution contained 1 mM CaCl_2_. Images were acquired at x60 magnification in line-scan mode (xt) at 0.5 kHz by confocal Nikon A1R microscope. Each cell was scanned along a longitudinal line and #10 xt frames (512 pxls x 512 pxls) were acquired. Non-cell fluorescence was acquired too to allow background fluorescence measurement. Confocal setting parameters were kept constant throughout experimental groups to permit group comparison analysis. Images were analysed by SparkMaster plugin (Fiji) software 42. Automatic spark detection threshold (criteria) was imposed to 3.8. Only in focus Ca^2+^ sparks (amplitude > 0.3) were considered to quantify their characteristics. In particular, the following spark parameters were measured: frequency (N of events/s/100μm), amplitude (ΔF/F0), full width at half-maximal amplitude (FWHM, μm), full duration at half-maximal amplitude (FDHM, ms) and decay time constant (τ, ms). Spark mass (spark amplitude*1.206* FWHM^3^) was also calculated as index of Ca^2+^ spark volume.

### Expression analyses of significant genes associated with SenCM

Cardiomyocytes transcriptomic data from single-nucleus RNA sequencing (snRNA-seq) were obtained from public repository Human Cell Atlas https://www.humancellatlas.org). Data were referring to Litviňuková et al 29. We used atrial- and ventricular-specific subset to derive expression profile of specific genes. Data were normalized and scaled through Serurat package for R environment (ver. 4.0.6). Analysis was performed with Seurat package and heatmaps of the expression levels of senescence-associated genes were visualized using ggplot2 package on R environment. Heatmaps display the average level of expression at single cell level (colour scale 0-1) and frequency of expression (dots dimensions) in the specific cell population. To minimize the influence of pathological unknown constituents, we did not included data from patients presenting Diabetes or Hypertension.

### Statistical analysis

Data are expressed as the mean ± standard error of the mean (SEM) of independent experiments. The differences between groups were tested with unpaired t-test or one-way ANOVA analysis as appropriate. Post-hoc comparison between individual means was performed with Tukey test. A p-value < 0.05 was considered statistically significant. For the correlation between KCNH2 expression and age a linear regression analysis showing 95% confidence band of the best fit trend line, was performed.

## Results

### Sub-lethal concentrations of Dox induce cell cycle arrest in human iCMs accompanied by negligible cytotoxicity

We successfully generated three different cell lines of human iPS from cardiac somatic cells source that were reprogrammed to functional cardiomyocytes (iCMs) using a small molecules protocol as previously described 32, 43. The differentiation protocol reached an efficiency of about 90% (Supplementary Figure S1A/B). Analysis of single cell electrical activity by patch-clamp recording showed that 70% of differentiated cells had ventricular-like phenotype with typical AP morphology with a sustained plateau phase. Those cells were distinct from iCMs with triangular AP shape mostly recapitulating atrial-like phenotype that account for about 30% of differentiated cells (Supplementary Figure S1C).

**Figure 1:**
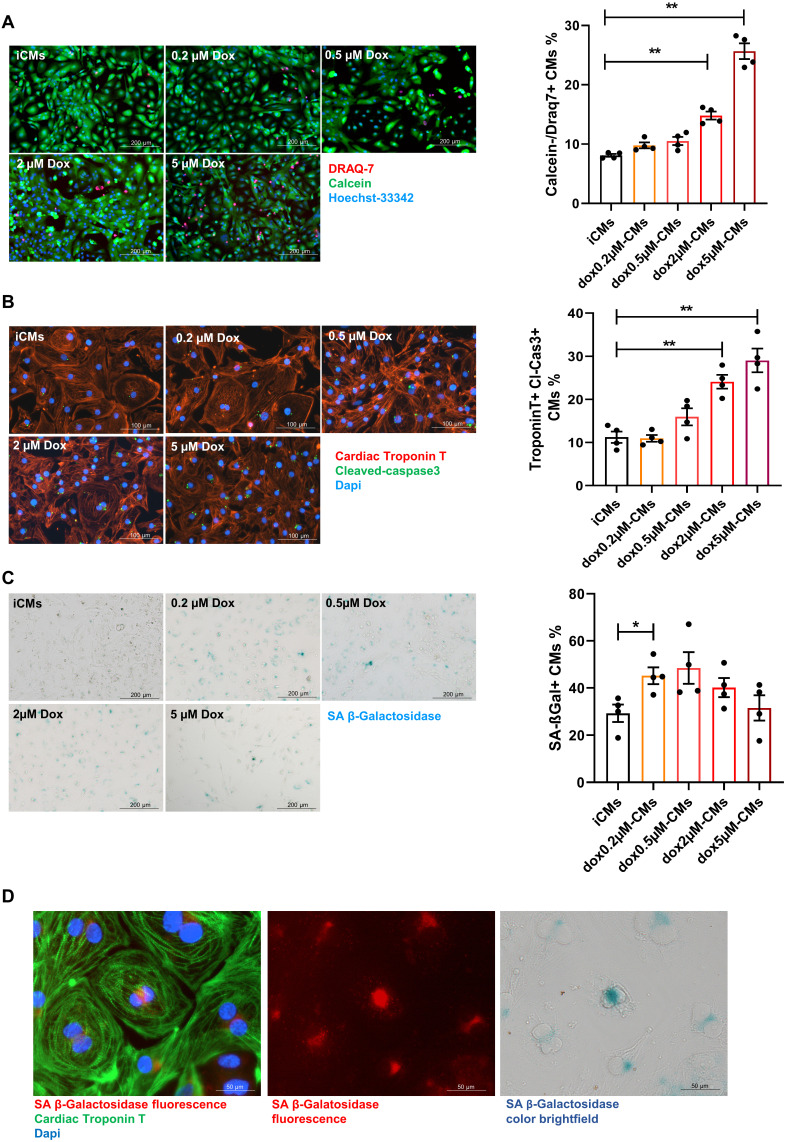
** Response of human iPS-derived cardiomyocytes (iCMs) to doxorubicin (Dox).** (A) Cell viability assay. Cells were labelled with Calcein-AM (green, viable cells) or DRAQ7 (red, dead cells) as described in methods section. Quantitative data (ratio of DRAQ7 positive and Calcein negative cells on the total number of cells) are shown in the bar graphs representing means of four independent experiments ± SEM. * P < 0.05, ** P < 0.01 *vs* iCMs. (B) Apoptosis was assessed by cleaved caspase-3 staining. Cleaved caspase was stained in green. Counterstaining of nuclei was performed with DAPI (blue). Cardiomyocytes were stained with cardiac Troponin T antibody (cTnT Red). Quantitative data (ratio of caspase positive cells on the total number of cTnT positive cells) are shown in the bar graphs representing means of four independent experiments ± SEM. * P < 0.05, ** P < 0.01 *vs* iCMs. (C) representative bright-field microscopy images of SA β-gal staining in iCMs and in Dox-treated cells at different concentration (0.2-5 μM). The percentages of SA β-gal-positive cells are shown in the bar graphs representing the mean of four independent experiments ± SEM. * P < 0.05 *vs* iCMs. (D) representative image showing SA β-gal-positive in SenCM upon excitation at 628 nm (far-red spectrum). Cardiac cTnT is shown in green, SA β-gal is shown in red correspondent bright-field microscopy images of SA β-gal staining is shown in the right panel.

Premature senescence was induced in iCMs by a short-time exposure (3 hours) to sub-lethal concentrations of Dox followed by drug washout (Supplementary Figure S1D). Cells were analyzed for the expression of SA-β-gal and simultaneously monitored for cell death hallmarks. Cell death (DRAQ7 positive cells) remained negligible upon exposure to Dox at concentration ranging from 0.2 to 0.5 µM whereas at higher doses cell-viability decreased and apoptosis (number of iCMs positive for Tunel and expressing cleaved caspases-3) increased in a dose-dependent manner (Figure 1A-B; Supplementary Figure S2A). The concentration of 0.2 µM significantly increased the expression SA-β-gal by 25%. Such increase remains stable at 0.5 µM and decreased over higher doses in concomitance with augmented cell death (Figure 1C). Since chromogenic beta-gal substrate generates a far-red-shifted fluorescent signal 44, we further co-localized SA-β-gal expression with cardiac specific protein and visualized its intracellular distribution by immunofluorescence analysis (Figure 1D). The dose of 0.2 µM Dox was selected as critical for inducing onset of senescence phenotype with minimal cell death. Thus, iCMs treated with 0.2 µM Dox are hereinafter referred to as senescent-like CMs (SenCMs). Unless otherwise specified, measurements were performed 4 days after Dox treatment.

### SenCMs display senescence-associated pathways

We performed a series of experiments aiming to assess whether SenCMs exhibit typical features of SIPS 45, 46.

**Figure 2:**
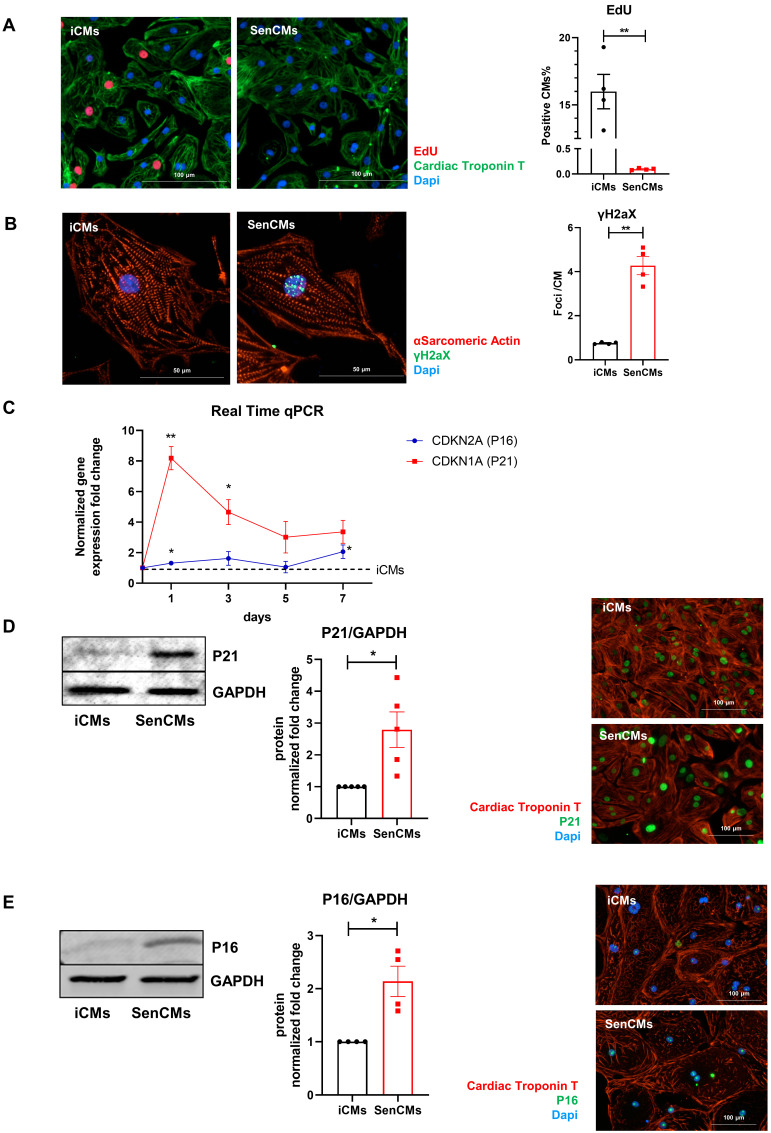
** Expression of senescence markers in SenCMs.** (A) EdU incorporation in iCM and SenCM. Cells were stained for cTnT followed by counterstaining with DAPI. Quantitative analysis of double-positive cells for EdU and cTnT is shown in the bar graphs representing four independent experiments ± SEM. * P < 0.05, ** P < 0.01 *vs* iCMs. (B) Representative images of immunofluorescence analysis of iCMs and SenCM using anti-γ-H2AX (green) and anti-sarcomeric α-actinin (red) antibodies. Cells nuclei were counterstained with DAPI. The number of γ-H2AX-positive foci per cell was calculated. Data are means of four independent experiments ± SEM. ** P < 0.01 *vs* iCMs. (C) Time-course for CDKN2A and CDKN1A mRNA relative expression in SenCMs versus iCMs (black dotted line). Data are means of four independent experiments ± SEM; * P < 0.05. (D) Western blotting analysis of p16 and p21 proteins in iCMs and SenCMs. Quantitative data are densitometry analysis of four independent experiments ± SEM; * P < 0.05. Images are showing nuclear expression of p16 and p21 (green) in iCMs and SenCMs stained for cardiac Troponin-T (red) and counterstained with DAPI.

**Figure 3:**
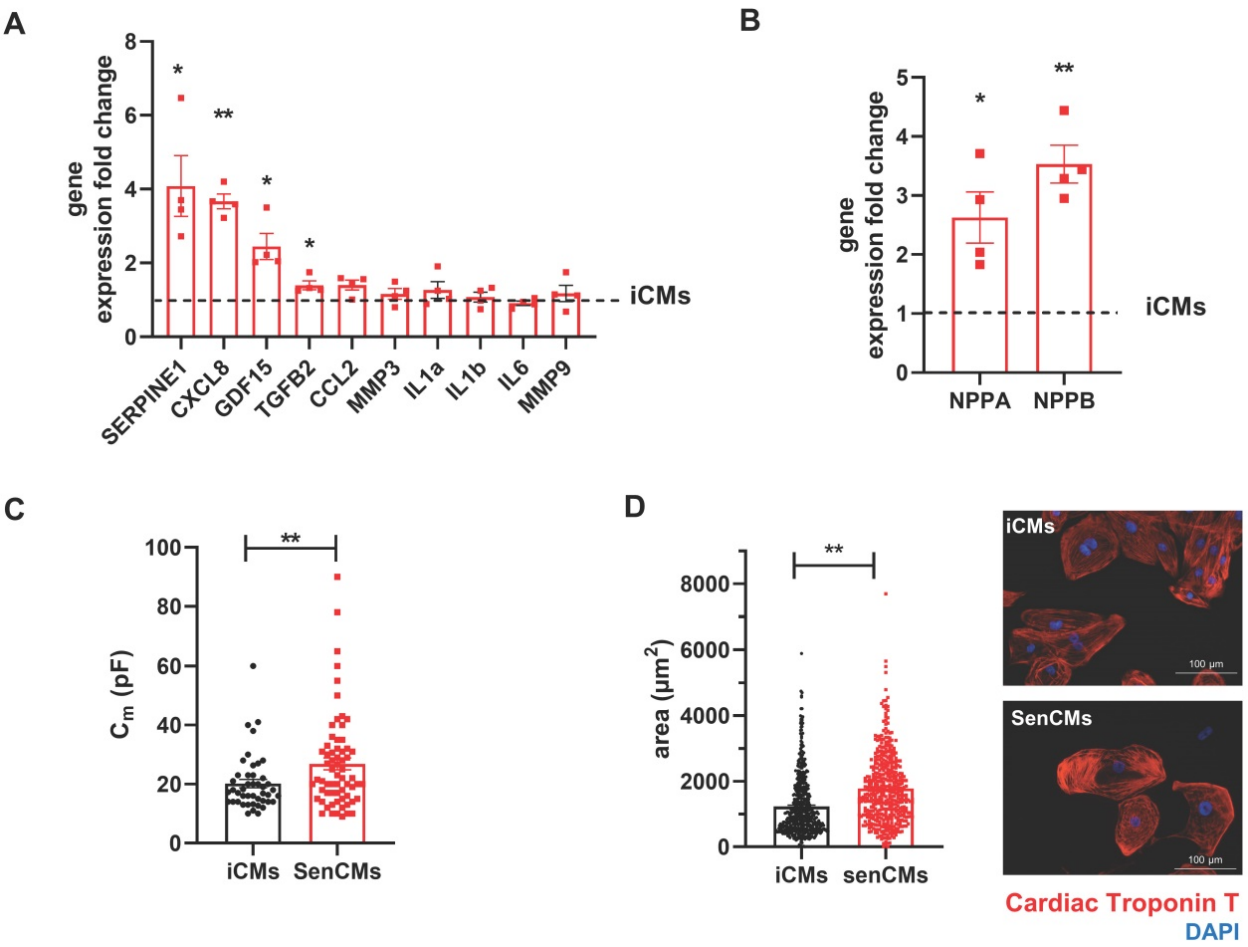
** Expression of SASP and induction of hypertrophy in SenCMs** (A) SASP-associated genes mRNA relative expression in SenCMs versus iCMs (black dotted line). Data are means of four independent experiments ± SEM. * P < 0.05, ** P < 0.01 *vs* iCMs. (B) NPPA and NPPB mRNA relative expression in SenCMs *versus* iCMs. Data are means of four independent experiments ± SEM. * P < 0.05, ** P < 0.01 *vs* iCMs. (C) Membrane capacitance (C_m_) recorded by patch-clamp technique from SenCMs and iCMs. Data are means of 65 and 45 cells respectively, pooled from four independent experiment ± SEM. * P < 0.05. (D) Cell surface area in SenCMs and iCMs. Data are means of 577 and 459 cells respectively, pooled from four independent experiment ± SEM. * P < 0.05.

Neonatal and adult CMs possess residual capability of DNA synthesis leading to poly-nucleation accompanied by limited cell division. New DNA-synthesis is also a feature of differentiated iCM and incorporation thymidine analogue 5-ethynyl-2′-deoxyuridine (EdU) account for about 15% of total cells in basal condition (Figure 2A). The number of dividing cells (AURKB positive) in such condition is about 0.9% (Supplementary Figure S2B). Thus, suggesting that EdU incorporation very likely reflects CM karyokinesis (without cytokinesis) during binucleation process 47, 48. In such context we assessed whether Dox affected the capability of iCMs to synthesize new DNA. We found that Dox treatment dramatically decreased the incorporation of EdU by 16-folds as compared to untreated iCMs (Figure 2A). In accordance with direct Dox-induced DNA damage, we observed a significant increase in the number of γ-H2AX-positive nuclear foci more than 5-fold in SenCMs compared to control iCMs (Figure 2B). Accumulation of γH2AX in SenCMs was associated with augmented expression of CDKN1A (hereafter p21^cip1/waf1^) and CDKN2A (hereafter p16^INK4a^) at both mRNA and protein levels (Figure 2C-E). Time-course RT-PCR experiments showed a pick of expression of p21^cip1/waf1^ at 24hrs after exposure to Dox and a subsequent decreasing overtime from day one to seven. p16^INK4a^ showed slight but constant increase overtime (Figure 2C). Most important p21^cip1/waf1^ and p16^INK4a^ were also increased at protein level as shown by WB and immunostaining at day 4 after exposure to Dox (Figure 2D-E). SenCMs were characterized by an increased expression of SASP-associated genes (Figure 3A). Transcripts of CXCL8, TGFB2, SERPINE1 and GDF15 genes were significantly overexpressed in SenCMs *vs* iCMs. Remarkably, levels of expression of CXCL8 and SERPINE-1 were 4-fold higher in SenCMs *vs* iCMs. Finally since aged hearts develop significant cardiac hypertrophy 49, we assessed whether this also occurs in our model. The expression of atrial natriuretic peptides (NPPA) and brain/B-type natriuretic peptides (NPPB), known to be predominantly expressed by the post-natal heart in response to stress-associated hypertrophy 50, were both significantly upregulated in SenCMs as compared to iCMs (Figure 3B). The occurred hypertrophy in Dox-treated CMs was also confirmed by measuring the cell membrane capacitance (C_m_) which reflects cellular dimensions (Figure 3C) and was further confirmed by direct measurement of CM surface-area (Figure 3D).

### Dox-induced senescence is associated with mitochondrial dysfunction and oxidative stress in SenCMs

Mitochondria are essential determinants of cellular homeostasis in CMs due to the high energetic demand of these cells. To gain insights into the Dox-induced mitochondrial impairment in SenCMs, we assessed key parameters of mitochondrial respiration by using a Seahorse extracellular flux analyzer.

**Figure 4:**
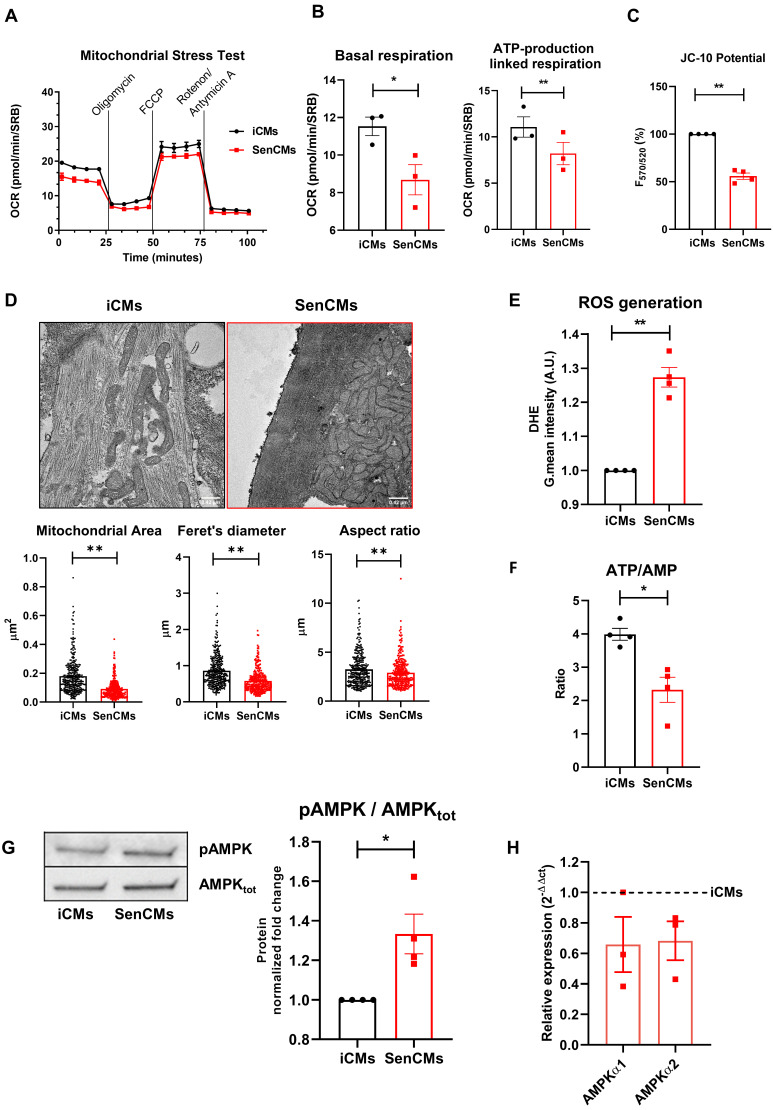
** Induction of mitochondrial damage in SenCMs.** (A) Measurement of oxygen consumption rate (OCR) by Seahorse-XF assay. Time-course of oxygen consumption rate (OCR) is shown in iCMs (black line) and SenCMs (red line). Timing for injection of Oligomycin 1µM, FCCP 0.5µM, Rotenone/Antimycin A 1µM OCR are indicated. (B) Basal respiration was measured as OCR level previous to Oligomycin treatment - minimum OCR level after Rotenone/Antimycin A injection. ATP linked production was measured as maximum OCR level after Oligomycin injection - Basal Respiration. Normalization for cell number was obtained with Sulforhodamine B assay. Data are means of three independent experiments ± SEM; * P < 0.05, ** P < 0.01 *vs* iCMs. (C) Mitochondrial membrane potential (ΔΨm) is evaluated by potential-sensitive dye JC-10. The graph represents the means of four independent experiments ± SEM ** P < 0.01 *vs* iCMs. (D) Representative TEM images and quantitative analysis (surface area, Feret's diameter and aspect ratio) of mitochondria morphology in SenCMs (N=136) *vs* iCMs (N=80). Data are pooled from three independent experiments ± SEM ** P < 0.01. (E) Reactive oxygen species were assessed in SenCMs *vs* iCMs using DHE assay. Cells were incubated with DHE for intracellular H_2_O_2_ detection as described in Methods and Materials. Quantitative data are shown in the bar graph as means of four independent experiments ± SEM; * P < 0.05, ** P < 0.01 *vs* iCMs. (F) ATP/AMP ratio was by measuring intracellular ATP and AMP levels as described in Methods and Materials. Data are means of four independent experiments ± SEM; * P < 0.05 vs iCMs (G) pThr172 AMPK:AMPK_tot_ protein expression levels. Quantitative data of four independent experiments ± SEM (densitometric values for the proteins of interest normalized for GAPDH). (H) AMPKα1 and AMPKα2 mRNA relative expression in SenCMs versus iCMs. Data are means of three independent experiments ± SEM. * P < 0.05, ** P < 0.01 *vs* iCMs.

SenCMs showed decreased OCR at basal respiration phases, with significant decrease in ATP-production linked respiration (Figure 4A-B). A loss in mitochondrial membrane potential in SenCMs was evaluated by JC-10 assay (Figure 4C). Membrane potential (ΔΨm) of mitochondria became significantly depolarized in SenCMs (Figure 4C), suggesting a potential disruption in organelle integrity. At ultrastructural level SenCMs showed significant mitochondrial perturbations that were characterized by irregular architecture with loss of defined cristae and changes in general shape with significant decreased surface area, Feret's diameter and aspect ratio, indicating a longitudinal shortening (Figure 4D). Moreover, a clear mitochondrial intracellular clustering was observed in SenCMs, suggesting an altered mitochondrial relocation. Mitochondrial dysfunction occurring during senescence process is generally associated with increased production of ROS 51. Indeed, SenCMs had significantly increased in ROS levels as compared to iCMs (Figure 4E) and generalized decline of metabolic capacity, as shown by the significant decrease in the cellular ATP/AMP ratio (Figure 4F). The imbalance in the energetic fluxes in SenCMs was associated with increased activity of the 5ʹ-AMP-activated protein kinase (AMPK) as highlighted by the positive ratio between the Thr172 phosphorylated AMPK (pAMPK) and the total protein level (Figure 4G). However, the increased activity was not accompanied by concomitant increase in mRNA expression level (Figure 4H). This suggests that cytosolic AMP levels could intrinsically increase AMPK activity by inducing its allosteric activation 52.

### Dox-induced senescence affects electrical properties and intracellular Ca^2+^ dynamics of SenCMs

**Figure 5:**
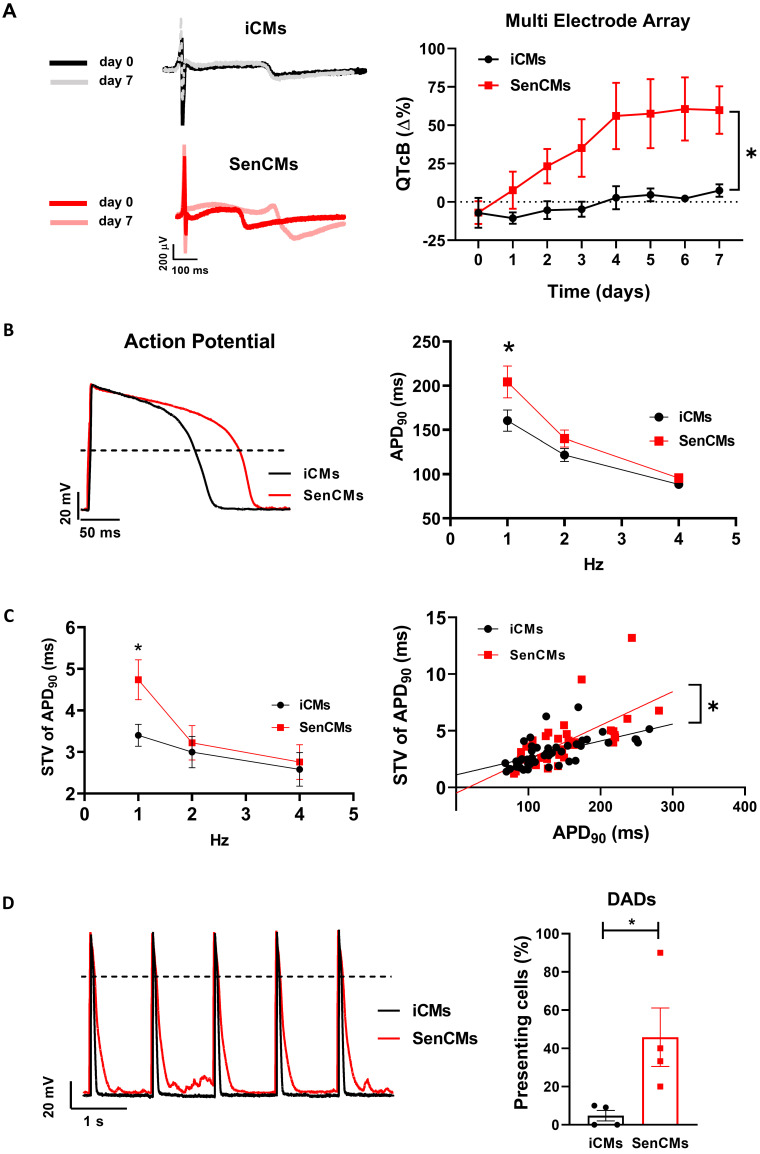
** Alterations of electrical activity in SenCMs.** (A) The electrical activity of spontaneously beating clusters of iCMs and SenCMs was recorded using MEA for 7 consecutive days after Dox treatment. Data are means of five independent experiments ± SEM; * P < 0.05 vs iCMs. QTcB: QT-interval corrected by Bazett's formula. Examples of field potentials in iCMs and SenCMs at day 1 and 7 are shown. (B) Rate-dependency of APD in isolated SenCMs (N=17) vs iCMs (N=22). Data are pooled from four indipendent experiments. ± SEM. * P < 0.05. Representative APs recorded at 1 Hz in each group are shown. Numerical I_K1_ (see Methods) was injected through Dynamic Clamp technique to compensate the low expression of native I_K1_ in iCMs. (C) Rate-dependency of short term variability (STV) of APD_90_ (left panel) and linear correlations between STV of APD_90_ and APD_90_ values (right panel) in each group; data from all stimulation rates were pooled. Data are means of at least 20 cells for every group, pooled from four independent experiments ± SEM. * P < 0.05. (D) DADs incidence in each group; recordings at 1Hz in each group are superimposed on the left to highlight DADs occurrence. *p<0.05 *vs* iCMs.

Aging is associated with an increased incidence of cardiac arrhythmias and heart failure. Indeed, the ability of SenCMs to respond to stressors is compromised by ionic remodelling, abnormalities in Ca^2+^ handling, and defective SR-mitochondria crosstalk 53, 54. Dox is also known to cause arrhythmias in humans as well as animal models 55-57. Using the MEA technology, we sought to explore whether Dox-induced senescence may affect FPD in spontaneously beating clusters. We found that electrophysiological properties of multicellular preparations of SenCMs were significantly altered as compared to iCMs, with a time-dependent increase of the corrected FPD (named QTcB) (Figure 5A). Notably, the prolongation of QTcB worsened over time and reached a steady-state value 5-7 days after single Dox exposure at day 0.

As the QT interval reflects the duration of the AP, we further investigated by patch-clamp analysis the specific contribution of inward and outward ionic currents that might explain the QT prolongation observed at multicellular level. As first attempt we measured at a single-cell level the electrical activity in terms of the duration of the AP, measured at 90% of repolarization (APD_90_). Both iCMs and SenCMs well adapted in terms of APD shortenings as the stimulation frequency increased; however, at low stimulation rate (1Hz), SenCMs showed a huge APD prolongation in comparison to iCMs (Figure 5B). As the increase in APD_90_ is directly linked with the variability of repolarization phase, we assessed the rate-dependency of STV APD_90_. As expected, STV resulted increased in SenCM at 1 Hz (Figure 5C) as a direct consequence of the prolongation of APD_90_. Moreover, the linear correlation STV/APD_90_ was steeper in SenCMs than iCMs, suggesting that other Dox-induced effects in addition to APD_90_ prolongation can affect the variability of APD. Overall, these results indicate that SenCMs have a pronounced tendency to arrhythmogenesis in comparison to iCMs. This was further confirmed by the higher number of cells exhibiting DADs among the treated cells as respect to iCMs (Figure 5D).

**Figure 6:**
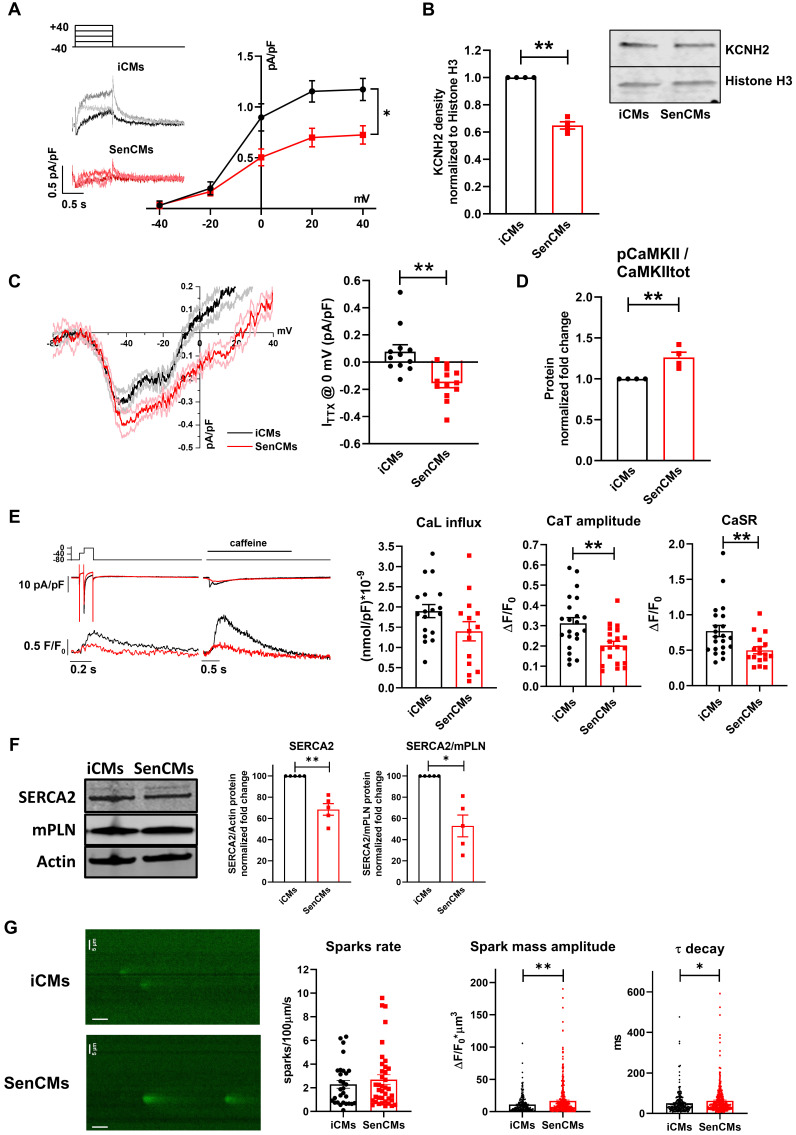
** Alterations of I_Kr_, I_NaL_ and Ca^2+^ handling in SenCMs.** (A) E-4031 (3µM)-sensitive currents (I_Kr_) activated according to the protocol shown on top and relative I/V relationships in iCMs (N = 21) and SenCMs (N = 26) 5-7 days after treatment. Data pooled from four independent experiments are presented as ± SEM. * P < 0.05, ** P < 0.01 *vs* iCMs. (B) HERG (KCNH2) protein expression levels in iCMs and SenCMs. Quantitative data of four independent experiments ± SEM (densitometric values for the protein of interest normalized for histone H3). ± SEM. * P < 0.05, ** P < 0.01 *vs* iCMs. (C) TTX (2 µM)-sensitive current (I_TTX_) activated during slow voltage ramps (28 mV/sec) from a holding potential of -100 mV. Mean ± SEM I/V relationships for iCMs (N = 12) and SenCMs (N = 13) are shown. Data pooled from four independent experiments are presented as ± SEM. * P < 0.05, ** P < 0.01 *vs* iCMs. Statistics of I_TTX_ at 0 mV, representative of I_NaL_, are reported on the right. (D) pThr286 CAMKII:CAMKII total protein expression levels in SenCMs versus iCMs. Quantitative data are from four independent experiments ± SEM (densitometric values for the proteins of interest normalized for GAPDH). * P < 0.05, ** P < 0.01 *vs* iCMs. (E) Membrane currents and Ca^2+^ transients (CaT) were recorded simultaneously according to the voltage clamp protocol shown on top in Fluo4-loaded iCMs. Examples (left panel) and statistics (right panel) of CaL influx, CaT amplitude and caffeine-induced CaT amplitude (estimating CaSR) in iCMs (N = 22) and SenCMs (N= 14) 5-7 days after Dox treatment. Data pooled from four independent experiments are presented as ± SEM. * P < 0.05, ** P < 0.01 *vs* iCMs. (F) SERCA2 and monomeric (m) PLN protein expression levels in iCMs and SenCMs. Quantitative data of five independent experiments ± SEM (densitometric values for the protein of interest normalized for actin). (G) Statistics of Ca^2+^ spark rate and characteristics in SenCMs (N=410) *vs* iCMs (N=266). Line scan (xt) images are shown on the left (time bar: 100 ms). * P < 0.05, ** P < 0.01 *vs* iCMs.

We investigated the rapid delayed rectifier K^+^ current (I_Kr_) I/V relationship, mediated by the HERG (also known as KCNH2) channel, as possible mechanism responsible for reduced repolarization reserve in SenCMs. SenCMs showed significant downregulation of I_Kr_ density in comparison to control iCMs (Figure 6A). Moreover, the protein expression level of the HERG channel at this time point was reduced in SenCMs *vs* iCMs (Figure 6B). As it was the first time that a decrease in expression of KCNH2 was associated with senescent CMs, we took advantage of our in-house biobank of human atrial appendage tissue specimens to verify a possible correlation between the protein levels and the age of the donors. Consistent with the *in vitro* data, we found a significant inverse correlation between KCNH2 expression and age of the patient (Supplementary Figure S3A).

A further cause of prolonged QT in CMs might be ascribable to an increased inward current during the plateau phase of the AP. Thus, we evaluated the Dox-induced modulation of the late component of Na^+^ current (I_NaL_), a good candidate accordingly to its sensitivity to the cellular redox state 58. I_NaL_ I/V relationships were obtained by measuring TTX (2 µM)-sensitive current (I_TTX_) during slow voltage ramps. Inward steady-state I_TTX_ might be representative of both Na^+^ window current at negative potentials and I_NaL_ at more positive potentials 41. While peak I_TTX_ at -39 mV (mostly representing I_Naw_) was not significantly affected in SenCMs, I_TTX_ measured at 0 mV (mostly representing I_NaL_) significantly increased in SenCMs compared to iCMs (Figure 6C). Accordingly, the proportion of CAMKII phosphorylated on the Thr286 of its alpha subunit (pCAMKII), a well-known modulator of I_NaL_ 59, increased in SenCMs (Figure 6D). Overall, the combination of I_Kr_ downregulation and I_NaL_ enhancement may largely justify the Dox-induced senescence-dependent electrical remodeling leading to QT/APD prolongation.

Next, to complete the characterization of SenCM phenotype, we studied the intracellular Ca^2+^ dynamics in FLUO-4 voltage clamped iCMs (Figure 6E). In comparison to controls, Ca^2+^ transient (CaT) amplitude and sarcoplasmic reticulum Ca^2+^ content (CaSR), evaluated through a caffeine (10 mM) pulse, were significantly reduced in SenCMs 5-7 days after treatment, leading to a similar fractional release (0.41±0.04 *vs* 0.44±0.04, ns). As expected, SR Ca^2+^ levels evaluated by measuring caffeine-induced CaT amplitude (Figure 6E) or caffeine-induced I_NCX_ (Supplementary Figure S4A) showed comparable results. Both Ca^2+^ influx through L-type Ca^2+^ channel and peak ICa_L_ density (-9.8±1.4 pA/pF *vs* -11.1±1.1 pA/pF, ns) at 0 mV were not significantly affected in SenCMs. SERCA2 protein levels were significantly reduced in SenCMs, while the protein levels of the physiological SERCA2 inhibitor phospholamban (PLN) in both the monomeric (m) and pentameric forms were not altered, leading to a SERCA2/PLN ratio reduction in SenCMs (Figure 6F), a finding previously reported in aged animal models^60^. To have a complete overview of SR function in SenCMs, potential alterations in SR Ca^2+^ release in addition to SR Ca^2+^ uptake, was evaluated in SenCMs by measuring Ca^2+^ sparks rate and characteristics. While Ca^2+^ sparks rate was not significantly altered in SenCMs in comparison to iCMs, analysis of Ca^2+^ sparks parameters underlined spatial and temporal Ca^2+^ spark duration enhancement in SenCMs, leading to a greater global volume of SR Ca^2+^ released by each Ca^2+^ release unit (namely spark mass) (Figure 6G and Supplementary Figure S4B). This finding well fits with the increased CaMKII activity in SenCMs, known to increase SR Ca^2+^ leak 61. Moreover, accordingly to SERCA2 depression, Ca^2+^ sparks decay became slower (that is τ decay enhancement) in SenCMs (Figure 6G). Overall, these data suggest a huge alteration of intracellular Ca^2+^ dynamics that largely explain the proarrhythmic phenotype of SenCMs (i.e. DADs occurrency).

### Model comparison with aging mice CMs

**Figure 7:**
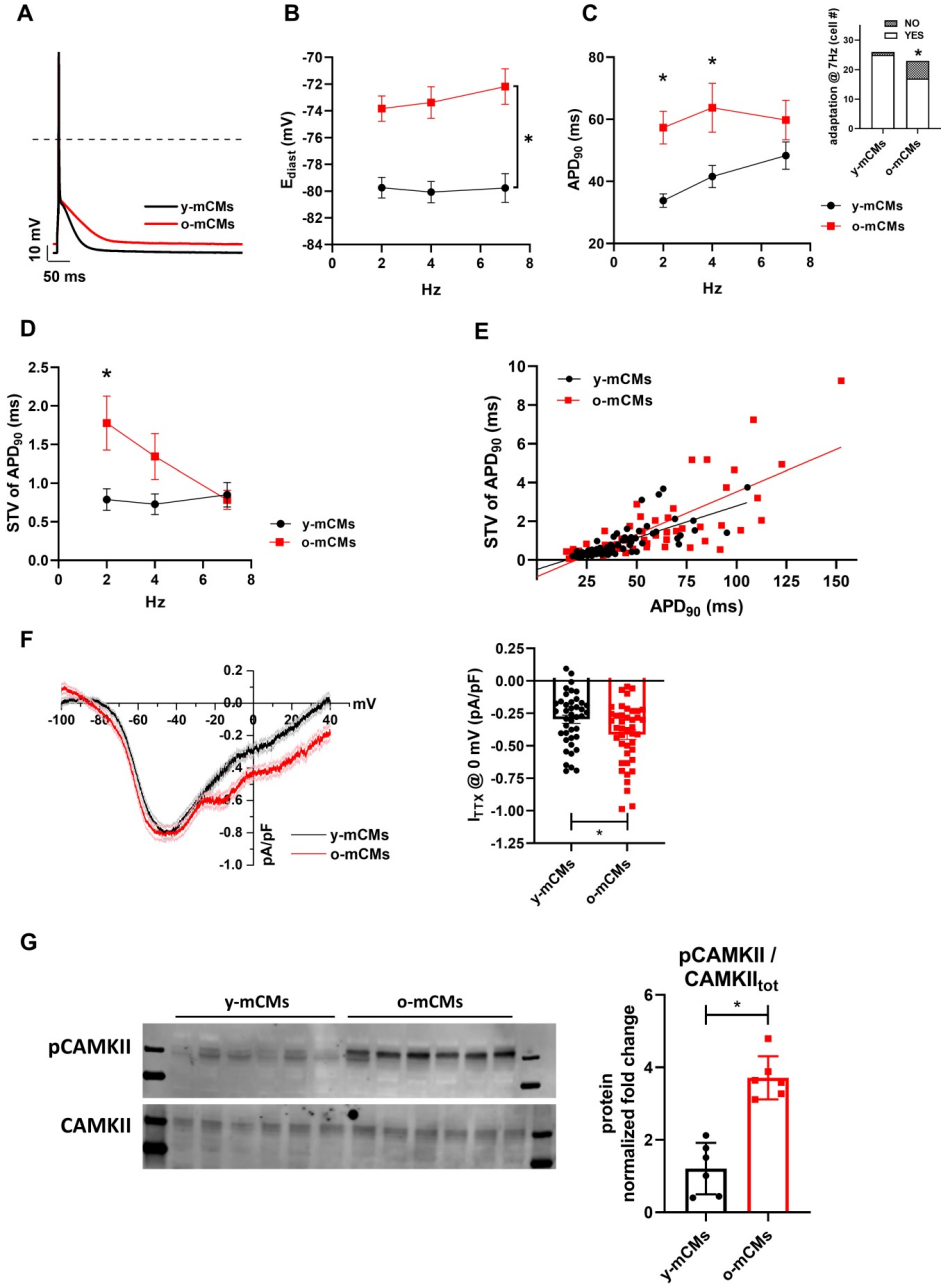
** Validation of Dox-induced senescence through physiological senescence in mice**. (A) APs recorded at 2 Hz in old (o-) *vs* young (y-) mCMs are superimposed. (B-C) Statistics of rate-dependency of E_diast_ and APD_90_ in y-mCMs (N=25-34) and o-mCMs (N=17-31). In comparison to y-mCMs, a significative portion of o-mCMs failed to adapt at 7 Hz (statistics in the inset on the right). Data pooled from four independent experiments are presented as ± SEM; * P < 0.05, ** P < 0.01 *vs* iCMs. (D) Rate-dependency of short term variability (STV) of APD_90_. (E) linear correlations between STV of APD_90_ and APD_90_ values in each group; data from all stimulation rates were pooled. Data pooled from four independent experiments are presented as ± SEM; * P < 0.05, ** P < 0.01 *vs* iCMs. (F) TTX (2 µM)-sensitive current (I_TTX_) activated during slow voltage ramps (28 mV/sec) from a holding potential of -100 mV. Mean ± SEM I/V relationships for y-mCMs (N = 41) and o-mCMs (N = 42) (left panel) and statistics of I_TTX_ at 0 mV, representative of I_NaL_ (right panel). Data pooled from four independent experiments are presented as ± SEM; * P < 0.05, ** P < 0.01 *vs* iCMs. (G) pThr286 CAMKII:CAMKII total protein expression levels in o-mCMs in comparison to y-mCMs. Quantitative data are from six independent experiments ± SEM (densitometric values for the proteins of interest normalized for GAPDH).

To exploit potential specie-specific differences with mouse CMs, key electrophysiological features were also evaluated in CMs isolated from young (y-mCMs) *vs* old mice (o-mCMs) (Figure 7). o-mCMs exhibited a depolarized E_diast_ at all stimulation rates (Figure 7B) and prolonged APD_90_ at 2 and 4 Hz (Figure 7C). Moreover, a significant number of o-mCMs failed to adapt at higher stimulation rates (7 Hz) (inset Figure 7C). Of note, as expected, the rate-dependency of APD_90_ in mice shows opposite trend as compared to human cells. In line with human model, in o-mCMs the STV of APD_90_ was significantly increased at low stimulation rates (Figure 7D) and the STV/APD_90_ correlation tended to be steeper in comparison to y-mCMs (Figure 7E). As observed in SenCMs, the APD prolongation in o-mCMs was largely dependent on enhanced I_NaL_ (Figure 7F) that contextually was ascribable to the increased proportion of CAMKII phosphorylated on Thr286 (Figure 7G).

### Model validation using heart-tissue transcriptomic data

To put in scale our human-based in-vitro model of iCMs with naturally aging CMs, data from repository-available snRNA seq was used to validate age-related expression of key genes that were found up-regulated in SenCMs. In-line with our model, CDKN1A (p21^cip1/waf1^) and CDKN2A (p16^INK4a^) were significantly upregulated in aging heart with marked overexpression of the former. Similar trend was also found for GDF15, CCL2 and NPPA, NPPB in ventricular and atrial CMs respectively (Supplementary Figure S3B).

### Model validation using pharmacological approach

**Figure 8:**
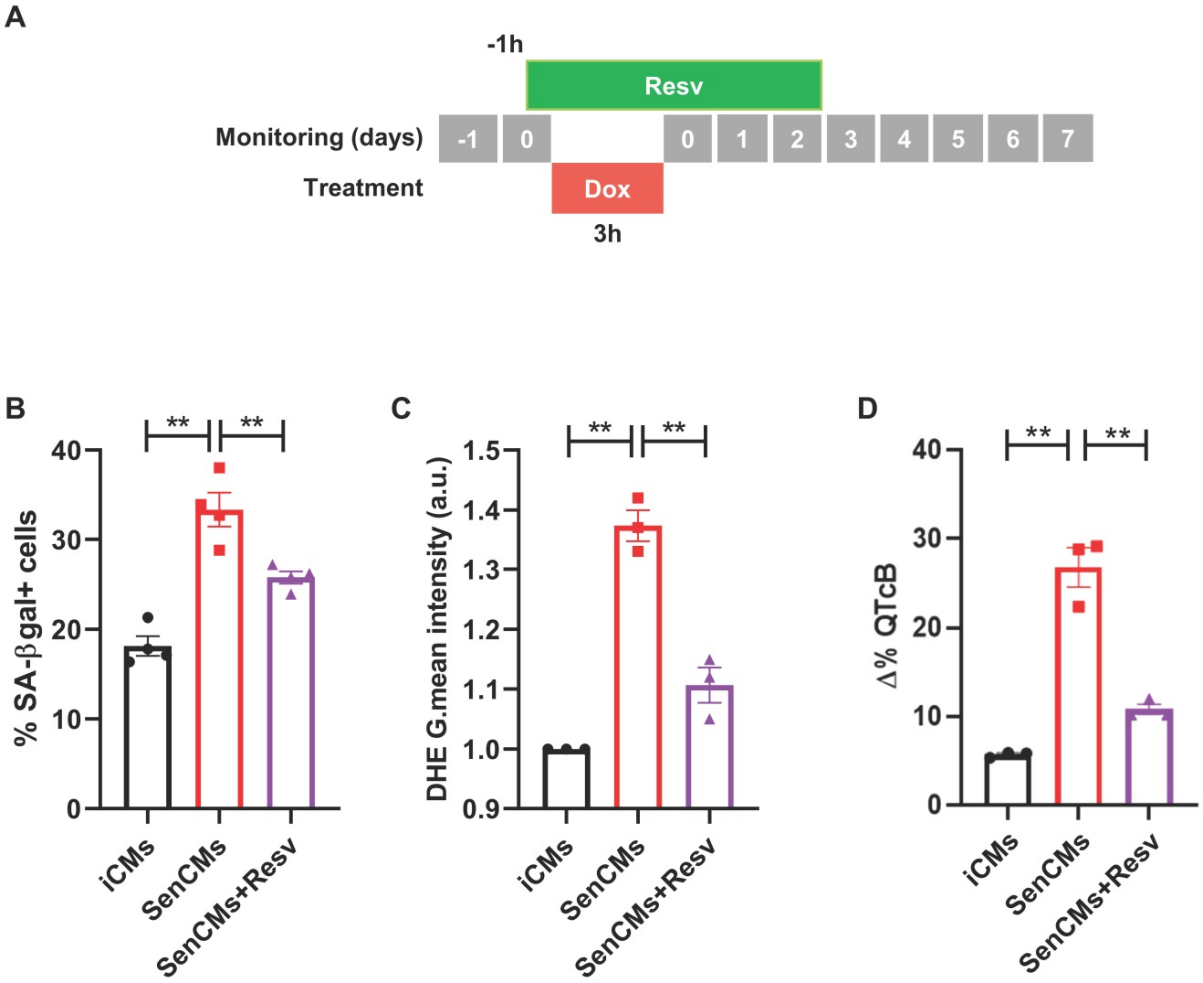
** Prevention of most aging markers by resveratrol treatment.** (A) Protocol outline. (B-D) Statistics of SA β-gal positive cells, ROS levels through DHE assay, and QTc prolongation in all experimental groups (iCMs, SenCMs and SenCms + Resv 25 µM). Quantitative data of three-four independent experiments ± SEM. * P < 0.05, ** P < 0.01 *vs* SenCMs.

To validate our model as drug testing platform 62, 63 we treated SenCMs with a well-known antioxidant and anti-aging compound: Resveratrol (Resv) (Figure 8). SenCMs treated with Resv showed significant reduction in SA-β-gal expression as well as in ROS production as compared to its counterpart. Notably at functional level, Resv was able to rescue prolongation of QTcB to basal level. All together, these data showed that our model is a reliable cardiac-specific platform for testing potential senolytic and/or senostatic agents.

## Discussion

Stressors such as DNA-damaging agents 64, overexpression of oncogenic genes 65, and oxidants 66 can induce age-independent senescence. SIPS not only plays a role in the arrest of cell cycle and cell propagation in replicating tissues 67, but also impairs the functionality of organs with scarce residual replicative capacity, as it is the heart. SIPS is considered a novel mechanism in the heart remodelling after acute myocardial infarction 27, 68, cancer therapy-induced cardiotoxicity 28 and long-term cardiovascular complications following sepsis 12. Maejima et al. reported that murine neonatal CMs that undergo SIPS have common features of replicative-senescence, including upregulation of cell-cycle regulatory inhibitors, expression of SA-β-gal, and the attenuation of telomerase activity 23. Here we extended existing data to human iCMs, overcoming potential specie-specific limitations, and exploring for the first time functional electrophysiological properties of aged human CMs. The present report showed that sub-toxic concentration of Dox induced expression of both p21^cip1/waf1^ and p16^INK4a^ in human CMs and caused cellular senescence. Senescent cells displayed components of DNA-damage response (DDR), such as γH2AX-positive foci. Possible consequence of this effect is that the residual CM renewal - which has been demonstrated occurring in humans by approximately 0.5% to 2% per year 69 and accounting for a stable turnover of CMs in the healthy adult heart 70, might be impaired by SIPS. In a broader view, here we showed evidence that underlying mechanisms of diminished cardiomyocyte turnover with aging 69, 71 are likely attributable to cardiomyocyte senescence. In fact, the synthesis of new DNA was dramatically abrogated in SenCMs. SenCMs well recapitulate features of senescence cells in terms of “non-canonical senescence-associated secretory phenotype” as shown by Anderson et al 72. The latter may contribute to age-related cardiac dysfunction as SenCM-associated SASPs including GDF15 and TGFB2 have been associated with myofibroblast activation and CMs hypertrophy 72.

Finally, SenCMs showed mitochondrial damage in terms of a depolarized mitochondrial membrane potential, impaired energy production capability and ultrastructural disruption, that have been previously seen as effects of aging on mitochondria during the development of heart failure 73. Moreover, a clear mitochondrial intracellular clustering was observed in SenCMs, suggesting an altered mitochondrial relocation. Such “clustering” of intracellular organelles is coupled with β-gal signal that was mostly distributed in the peri-nuclear area (Figure 1D), which is consistent with retrograde transport along the microtubule system of intracellular organelles, including lysosomes, that have been previously described in aging CMs 74. A second possible explanation of mitochondrial accumulation is an occurring insufficient mitophagy in SenCMs. Under normal conditions, damaged mitochondria undergo asymmetrical fission where the dysfunctional mitochondrial fragments are then eliminated by mitophagy. Overall, the findings in this study support evidence in animal models, suggesting a defective clearance of damaged mitochondria 51.

Impairment of mitochondrial function has a direct consequence in increased level of ROS in SenCMs in line with evidences showing the involvement of ROS in cell senescence 75, 76. Interventions against ROS production and improving mitochondrial function represent attractive strategies to reduce age-related cardiac remodelling 76, 77. Consistent with this, iCMs treated with resveratrol prevented key aging hallmarks induced by Dox.

We provided evidence showing that senescence affects ionic currents in human CMs. We observed a reduction of the rapid delayed rectifier K^+^ current (I_Kr_) and enhancement of the late Na^+^ current (I_NaL_) in SenCMs *vs* control ones, resulting in a prolonged QTc interval. The latter constitutes a downright novel finding that supports at cellular level a recent population study by Rabkin and colleagues 78. They applied a rigorous analytical method to compare six QTc formulae and clearly showed that older individuals have a greater QTc. A significant association between QTc and age was evident in both sexes and regardless of whether they considered a linear or non-linear relationship 78. This finding was sustained by our data in human tissue showing a significant inverse relationship between age and KCNH2 expression. Although some aspects of the study from Rabkin and colleagues remain controversial as other investigators have reported no association between age and QTc 79, 80, our data supports the hypothesis that elderly patients are more vulnerable to situations and medications that might prolong the QTc, leading to increased propensity to arrhythmias.

The senescence-associated reduction of the repolarization reserve related to I_NaL_ enhancement in iCMs was well reproduced in old mouse CMs. The mechanisms underlying I_NaL_ enhancement can be related to changes in the cellular redox state and increased CAMKII activity 81 as we observed in both aging models. Accordingly to its rate-dependency, in both human and mouse models I_NaL_ enhancement affected APD especially at low rates, thus increasing temporal variability of repolarization, a well-known proarrhythmic condition 82. Indeed, even though I_NaL_ enhancement with aging might represent an inotropic support for the aged heart, abnormal I_NaL_ can contribute to the pathogenesis of both electrical and contractile cardiac dysfunction. Accordingly, we found that SIPS affects the pro-arrhythmogenic susceptibility expressed in terms of STV index; moreover, increased DADs incidence in SenCMs is symptomatic of a Ca^2+^ overload condition strictly dependent on intracellular Na^+^ and Ca^2+^ levels alterations. Although we confirmed the enhancement of the late Na^+^ current (I_NaL_) in CMs from old mice as compared to their young counterpart, we could not assess the contribution of I_Kr_ as such component of the repolarizing current is not expressed in mice 83, 84. Thus, highlighting the importance of having a “human-based” model to critical dissect specific mechanisms.

To analyse aging related functional changes in proteins involved in excitation-contraction (EC) coupling, Ca^2+^ handling measurements were performed in voltage clamped iCMs, thus, controlling membrane potential, any changes secondary to altered electrical activity were avoided. This approach was suitable to highlight in SenCMs SR Ca^2+^ content reduction, confirmed by a downregulated functionality of SERCA2 pumping activity. A similar finding has been reported in animal models 60 and can justify the diastolic relaxation impairment in the aged population 85.

Moreover, impairment of intracellular Ca^2+^ dynamics in SenCMs was underlined by measuring Ca^2+^ sparks characteristics and rate. SIPS induced increased Ca^2+^ spark mass, likely dependent on increased CAMKII activity 37. Thus, both increased SR Ca^2+^ leak and reduced SR Ca^2+^ uptake by SERCA2 contributed to depress SR Ca^2+^ content in SenCMs. Overall, alterations in intracellular Ca^2+^ compartmentalization in SenCMs can be considered a general hallmark promoting aging-induced electrical activity alterations (i.e. DADs incidence) 86. Ca^2+^ sparks characteristics reported in senescent cells from old mice and rats 87, 88 are not fully comparable to the present ones, probably because of species-specificity of the aging model. Thus, a “human-based” model of cardiac senescence is once again useful to better understand aging related mechanisms. We are aware that iCMs have some limitations. Important functional parameters such as the cells' resting membrane potential, the conduction velocities, and the amplitudes of the mechanical forces generated do not reach the same level of maturity as the adult CMs 89. This early maturity state represents a general limitation in the iPS-derived CMs and nowadays there are no available protocols that may completely overcome such limitation. Ongoing efforts are aiming to ameliorate the field and generate more mature CMs by optimizing extracellular matrix composition, mechanical and electrical training 90, 91. Nevertheless, our data sheds light on the significance of having a human *in vitro* platform for exploring cellular senescence in CMs in terms of pathogenic mechanisms and possible cardioprotective approach.

## Supplementary Material

Supplementary figures and table.
