# Supplementary material for: Stress-induced premature senescence is associated with a prolonged QT interval and recapitulates features of cardiac aging
Source: Theranostics. 2022 Jul 4;12(11):5237–57. doi: 10.7150/thno.70884 (PMC9274748; doi:10.7150/thno.70884)
Supplement: Supplementary file 1 — Supplementary figures and table. [file thnov12p5237s1.pdf]

Supplementary Material

Stress-induced premature senescence is associated with a prolonged QT interval and recapitulates features of cardiac aging

Edoardo Lazzarini[1#]; Alessandra Maria Lodrini[2#]; Martina Arici[2]; Sara Bolis[1,3]; Sara Vagni[2]; Stefano Panella[1]; Azucena Rendon-Angel[1,5]; Melissa Saibene[6]; Alessia Metallo[2]; Tiziano Torre[4]; Giuseppe Vassalli[3,5]; Pietro Ameri[7,8]; Claudia Altomare[1†]; Marcella Rocchetti[2†*]; Lucio Barile[1,5,9†*]

[1]Laboratory for Cardiovascular Theranostics, Cardiocentro Ticino Institute, Ente Ospedaliero Cantonale, Lugano, Switzerland; [2]Department of Biotechnology and Biosciences, Università degli Studi di Milano-Bicocca, Milano, Italy. [3]Laboratory of Cellular and Molecular Cardiology, Cardiocentro Ticino Institute, Ente Ospedaliero Cantonale, Lugano, Switzerland; [4]Department of Cardiac Surgery Cardiocentro Ticino Institute, Ente Ospedaliero Cantonale, Lugano, Switzerland; [5]Faculty of Biomedical Sciences, Università della Svizzera Italiana, Lugano, Switzerland. [6]Department of Earth and Environmental Sciences, Università degli Studi di Milano-Bicocca, Milano, Italy.[7]Cardiovascular Disease Unit, IRCCS Ospedale Policlinico, Genova, Italy.[8]Department of Internal Medicine, University of Genova, Genova, Italy. [9]Institute of Life Science, Scuola Superiore Sant'Anna, Pisa, Italy.

# The first two Authors contributed equally to the study, alphabetical order.

[†] These authors are senior authors

* Corresponding Authors

Running title: iPSC-derived cardiomyocytes as a platform for studying myocardial aging

Addresses for correspondence:

Lucio Barile, PhD
Cardiocentro Ticino Institute
Via Tesserete 48
6900 Lugano, Switzerland
+41 918053384
lucio.barile@eoc.ch

Marcella Rocchetti, PhD
University of Milano-Bicocca
Dept. of Biotechnology and Biosciences
P.za della Scienza, 2
20126 Milano, Italy
+39 0264483313
marcella.rocchetti@unimib.it

Supplementary Figure S1: (A) Representative images of cardiac specific markers cTnT (red), cTnI (green), a-actinin (red) in iCMs and SenCMs. (B) Quantitative data of eight independent experiments ± SEM. (C) Assessment of chamber-specific differentiation by APD morphology. Representative atrial-like (red) and ventricular-like (blue) APD shapes in iCMs are shown in the left panel. Quantitative data of three independent experiments ± SEM. * $P < 0.05$, *vs* atrial (AT). Right panel shows iCM culture representative of culture heterogeneity myosin light chain (red) and ventricular light chain (green) have been used as specific markers.

Supplementary Figure S2: (A) Apoptosis was assessed by Tunel assay. Nuclei of apoptotic cells were stained in green. Counterstaining of nuclei was performed with Hoechst (blue). Quantitative data (ratio of Tunel positive cells on the total number of nuclei) are shown in the bar graphs representing means of four independent experiments ± SEM. ** $P < 0.01$ *vs* SenCMs (B) Staining assessing cell division in cardiomyocytes. Aurora B Kinases staining (green), EdU incorporation (red), Cardiac Troponin T is stained in gray and nuclei are counterstained with DAPI. bar graphs representing means of four independent experiments ± SEM. ** $P < 0.01$ *vs* SenCMs

Supplementary Figure S3: (A) Linear regression analysis between KCNH2 expression in atrial tissue and age in thirteen different patients. The linear regression is showing the 95% confidence bands of the best fit trend line.

B data obtained from Human Heart Atlas Database were analysed stratifying patients into three age class (40-50 years old, 50-60 y.o., and 60-70 y.o.). Subset of Ventricular (left) and atrial (right) cardiomyocytes were considered for analysis. For every evaluated gene, percentage of cell expressing such gene is represented by dot size, while colour indicate normalized gene expression in accordance to the reported colour scale. Differential gene expression is statistically evaluated with Weighted Kolmogorov Smirnov (WKS) test followed by FDR correction, **$P < 0.05$.

Supplementary Figure S4: (A) SR Ca^{2+} content estimated through integration of NCX current elicited by caffeine (10 mM) in SenCMs (N=17) *vs* iCMs (N=22). nmol of Ca^{2+} entering the cell through NCX were normalized to cell capacitance. (B) Ca^{2+} sparks characteristics (amplitude, full width at half maximum FWHM, and full duration at half maximum FDHM) in SenCMs (N=410) *vs* iCMs (N=266). ** $P<0.01$ *vs* iCMs.

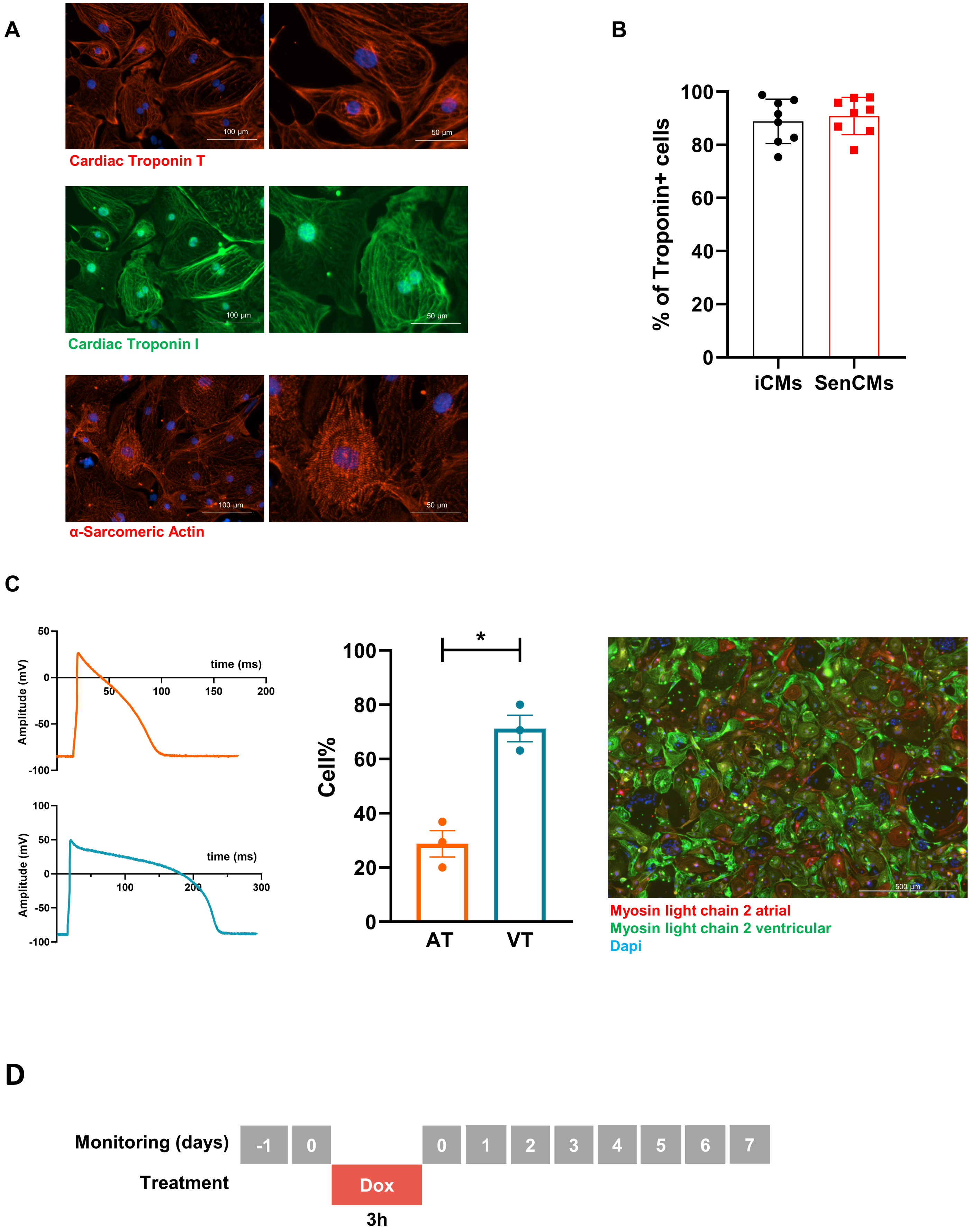

Supplementary Figure S1

A

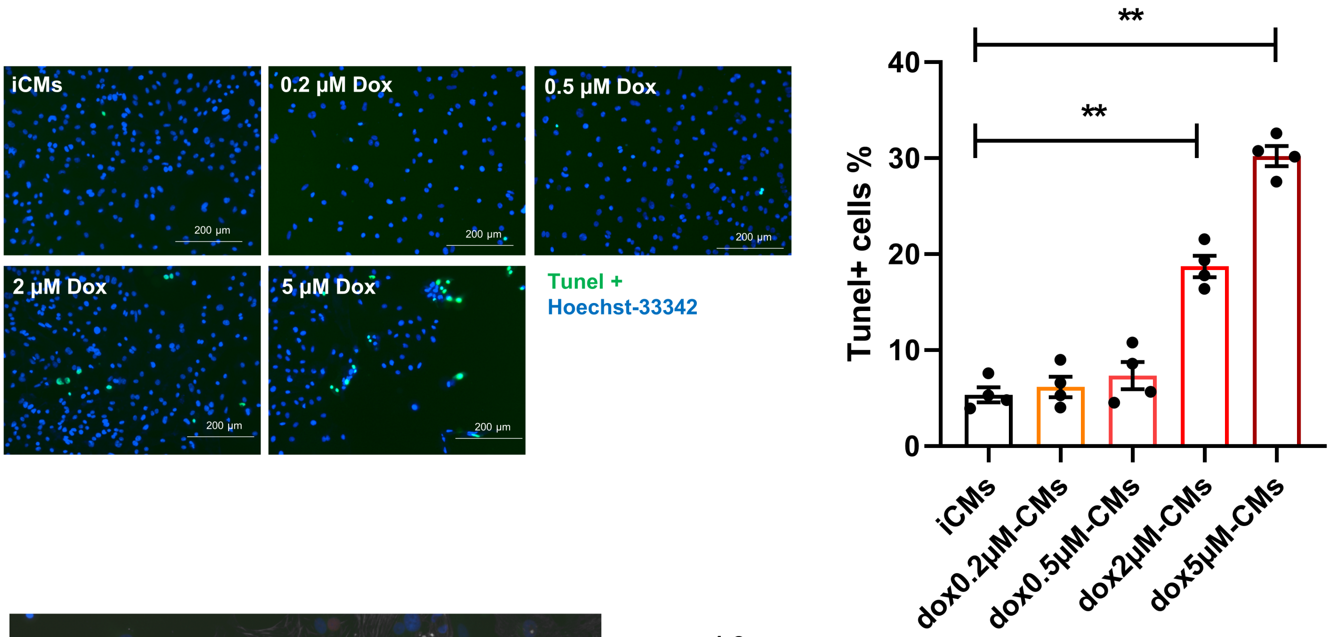

B

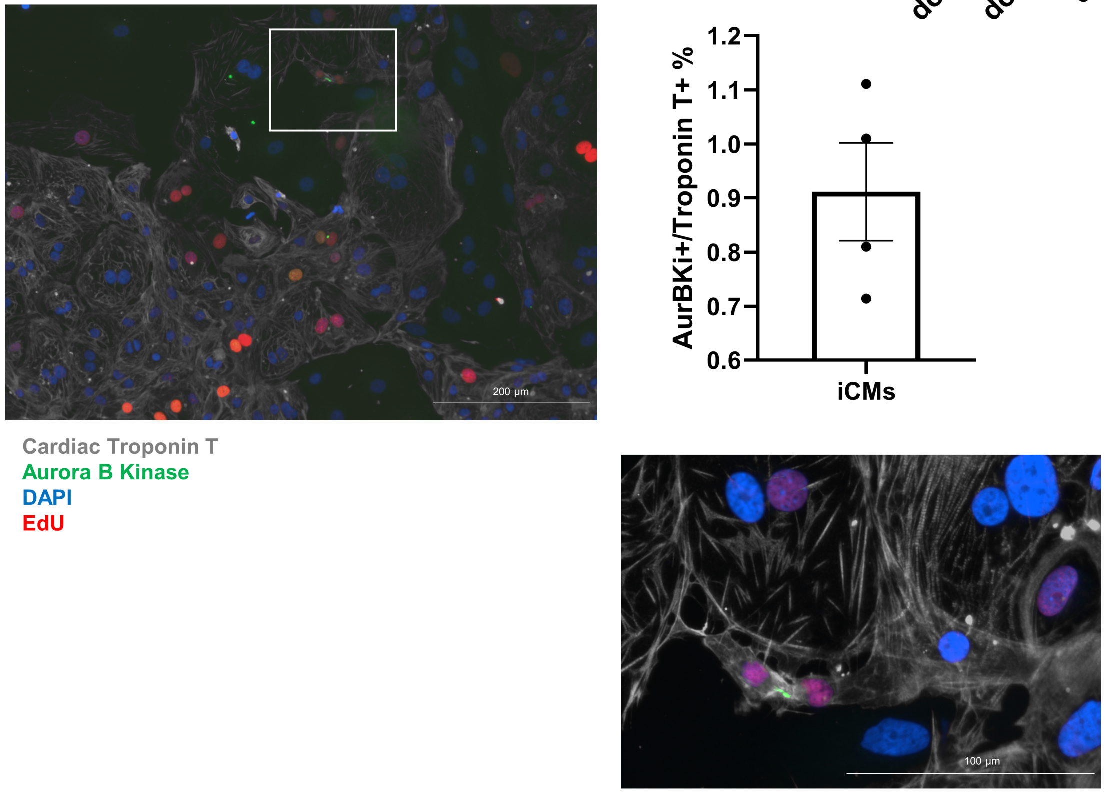

Supplementary Figure S2

A

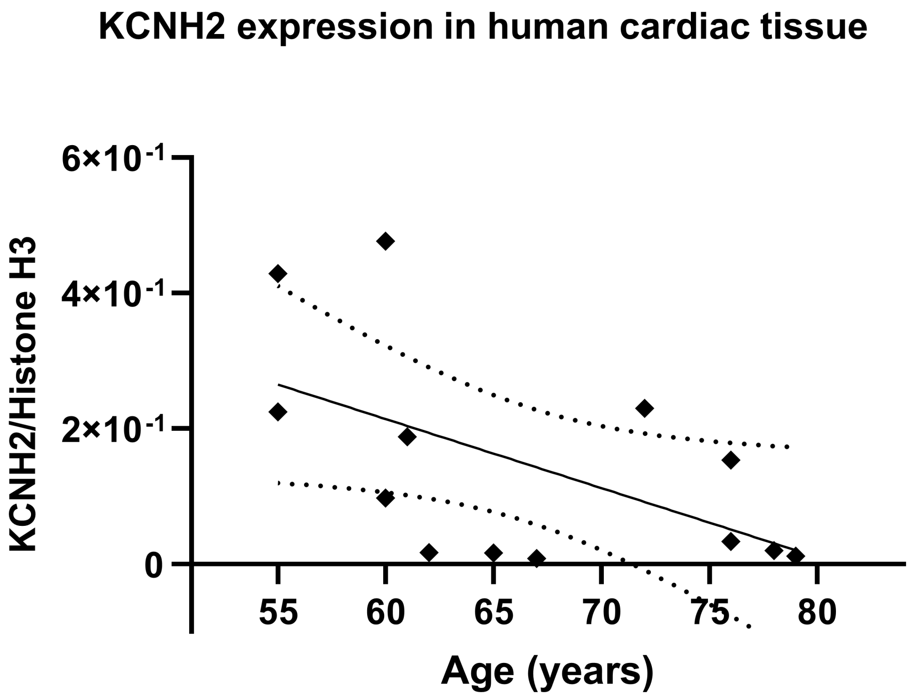

B

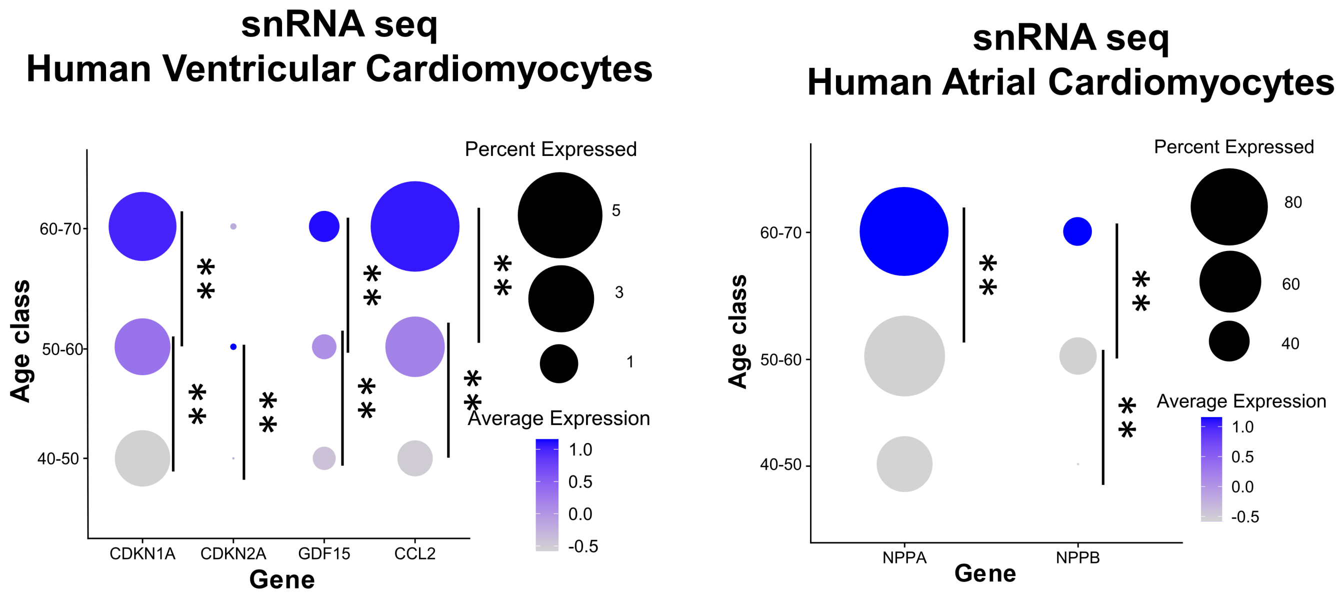

Supplementary Figure S3

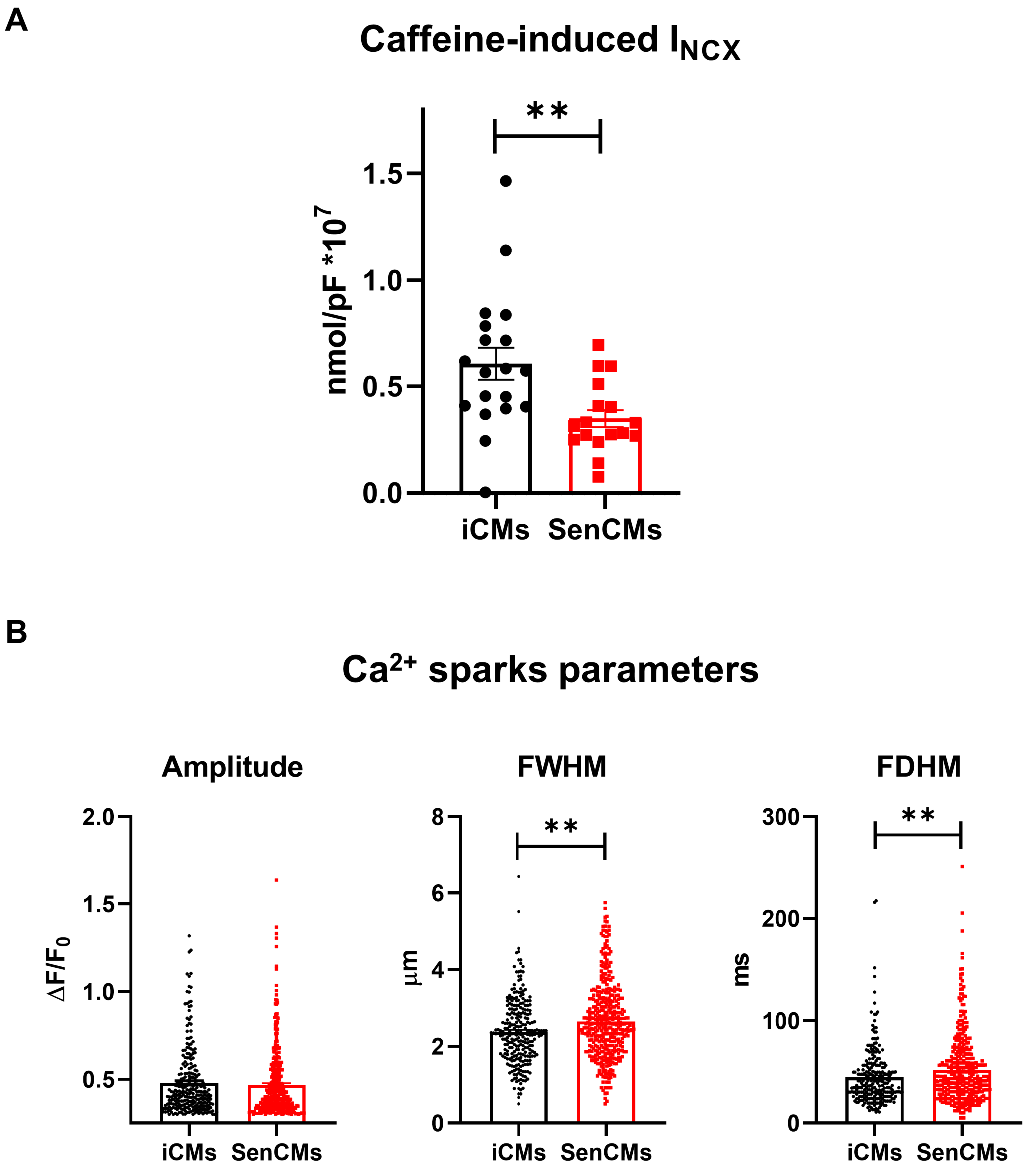

Supplementary Figure S4

Gene	F (5’-3’)	R (5’-3’)
**CDKN2A**	CTTCGGCTGACTGGCTGG	TCATCATGACCTGGATCGGC
**CDKN1A**	CACCTCACCTGCTCTGCTGC	GCTGGTCTGCCGCCGTTTT
**SERPINE1**	TTGCAGGATGGAACTACGGG	GTGGCAGGCAGTACAAGAGT
**CXCL8**	TCTGGACCCCAAGGAAAACTG	TCACTGGCATCTTCACTGATTCTT
**GDF15**	AGGTGAGAACCTTCTGGGGTT	CCTGGGAGTCTGTGCTTTTGG
**TGFB2**	CATCTACAACAGCACCAGGGA	CAACTGGGCAGACAGTTTCGG
**CCL2**	CCTTCATTCCCCAAGGGCTC	CTTCTTTGGGACACTTGCTGC
**MMP3**	TGAAATTGGCCACTCCCTGG	GGAACCGAGTCAGGTCTGTG
**IL1A**	CTTCTGGGAAACTCACGGCA	AGCACACCCAGTAGTCTTGC
**IL1B**	TTCGAGGCACAAGGCACAA	TTCACTGGCGAGCTCAGGTA
**IL6**	ATGAACTCCTTCTCCACAAGC	GAATCTTCTCCTGGGGGTACTG
**MMP9**	GCCACTACTGTGCCTTTGAGTC	CCCTCAGAGAATCGCCAGTACT
**NPPA**	TGAGCTTCCTCCTTTTACTGG	CCAGCAAATTCTTGAAATCCATC
**NPPB**	CTCCTGCTCTTCTTGCATCTG	TTGCGCTGCTCCTGTAACC
**AMPKA1**	ACAGCCGAGAAGCAGAAACA	TTGCCAACCTTCACTTTGCC
**AMPKA2**	TTGACAGGCCATAAAGTGGCA	TCGAACAATTCACCTCCAGACA
